# *Populus nigra* Bud Extract as a Standardized Alternative to Propolis: Evidence of Compositional Similarity—Functional Properties of an Oral Spray Containing *Populus nigra* Bud Extract

**DOI:** 10.3390/molecules31111836

**Published:** 2026-05-26

**Authors:** Luisa Mattoli, Andrea Lugli, Michela Burico, Giada Fodaroni, Denise Decarli, Mattia Gianni, Anna Maidecchi, Giulia Antonini, Silvia Tondi, Anna Gaetano, Valentina Fiordelli, Rita Pagiotti, Jacopo Lucci, Claudio Buttarini, Stefano Garetto, Raffaele Saladino, Donatella Pietrella, Valentina Mercati, Emiliano Giovagnoni

**Affiliations:** 1 Aboca S.p.A., Località Aboca 20, 52037 Sansepolcro, Italy; 2 Bios-Therapy, Physiological Systems for Health S.p.A., Località Aboca 20, 52037 Sansepolcro, Italy; 3 Department of Chemistry and Pharmaceutical Technologies, Sapienza University of Rome, Piazzale Aldo Moro n.5, 00185 Roma, Italy; raffaele.saladino@uniroma1.it; 4 Medical Microbiology Unit, Department of Medicine and Surgery, University of Perugia, Piazzale Severi, Building D, 4th Floor, 06129 Perugia, Italy; donatella.pietrella@unipg.it

**Keywords:** *Populus nigra*, bud extract, propolis, medical device, untargeted metabolomics, targeted metabolomics

## Abstract

*Populus nigra* buds contain resinous exudates rich in flavonoids, phenolic acids, terpenoids and other bioactive constituents. These exudates are the main botanical source of European Poplar-type propolis. Since hive-collected propolis shows strong botanical, geographical and hive contaminant variability, *P. nigra* bud resin exudate represents an attractive, standardizable and reproducible alternative for obtaining natural-complex ingredients. This study investigates the compositional relationship between Propolgemma^®^ standardized *P. nigra* buds (PBHE) and European propolis (PHE) hydroalcoholic extracts through integrated analytical approaches and evaluates the functional bioactivity of PBHE and a related oral spray formulation (Propolgemma^®^ spray forte, PBHE-SF). Untargeted metabolomic fingerprinting revealed clear clustering of *P. nigra* bud exudate with European propolis, demonstrating high compositional similarity. Targeted analyses confirmed that PBHE belongs to the poplar-type propolis family, while retaining additional bud-derived constituents such as salicylates, lignins and tannins, typical of bud tissue and largely absent from hive-collected propolis. Functionally, PBHE showed concentration-dependent antioxidant activity and significant inhibition of *Streptococcus pyogenes* biofilm at sub-MIC levels. PBHE, incorporated into a patented oral spray formulation (PBHE-SF), demonstrated strong mucoadhesion, high resistance to salivary wash-off, retention of antioxidant flavonoids on epithelial substrates and a mechanical barrier effect, reducing LPS-induced IL-6 release by 39%. It also showed dispersion of pre-formed *S. pyogenes* biofilms. PBHE emerges as a reproducible, plant-derived, bee-independent alternative to European propolis. Its chemical consistency, functional reliability, independence from bee foraging and from hive-derived contaminants improve the therapeutic potential on mucosal protection in medical device formulations and the suitability for scalable, controlled and industrially sustainable production.

## 1. Introduction

The genus *Populus* (Salicaceae) comprises deciduous tree species widely distributed across the Northern Hemisphere, particularly in temperate and boreal regions. It is divided into five taxonomic sections among which the *Aegiros* group includes *Populus nigra* L., the European black poplar native to Europe, Western Asia, and North Africa [1]. *P. nigra* is a pioneer species that thrives in alluvial habitats, with a straight trunk reaching up to 30 m and leaves of variable triangular to rhomboidal morphology.

Phytochemically, *Populus* species concentrate bioactive compounds primarily in the bark and buds. Traditional uses of *P. nigra* include the use for wound healing, alleviation of dermatitis symptoms, and the treatment of rheumatic and respiratory conditions, the buds being particularly valuated for their astringent and antiseptic properties [1,2]. The relevant organ for medical applications is the winter bud, harvested before break (February-March) [3]. The buds are subdivided into leaf and flower buds [3]. The leaf buds are elongated and rich in aromatic resin, whereas the flower buds are more globular and contain comparatively less resinous exudate. Their viscous exudate is a complex mixture of flavonoids (e.g., pinocembrin, galangin), phenolic acids and their esters (e.g., caffeic acid, ferulic acid, caffeic acid phenethyl ester), terpenoids and volatile aromatic compounds [2], providing multiple physiological and ecological functions such as thermal insulation, antimicrobial defence, insect repellence, and water retention.

Importantly, *P. nigra* bud resin exudate constitutes the principal botanical source of European propolis [1,4]; however, contributions from other *Populus* species (e.g., *Populus tremula*) and poplar hybrids have also been reported, in line with the intrinsic variability of propolis composition [4]. Propolis—the term derives from the Greek pro-polis, “in front of the city,”—is a resinous material collected by honeybees from plant tissue and mixed with wax and saliva to reinforce, seal and protect the hive [5,6]. Its chemical composition clearly indicates that propolis is fundamentally of plant origin [4], undergoing only minimal biochemical transformation by bees during collection and transport [7,8,9,10].

**Figure 1:**
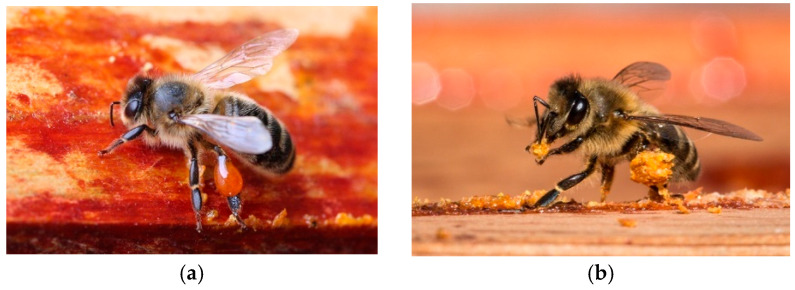
Bee-collected plant resin load (**a**) and propolis load (**b**) carried within the hind-leg pollen baskets (corbiculae) [12] (with the permission of Christopher Wren, drcwren@icloud.com).

The resin-gathering process is well documented and involves mandibular harvesting, transfer to the hind-leg corbiculae and subsequent mixing with wax inside the hive (Figure 1a,b) [11,12].

The botanical sources define the chemical composition of propolis, giving rise to several chemically and botanically distinct propolis types across geographic regions [4,13,14]. In this context, propolis composition has often been summarized using schematic representations of major constituent classes (e.g., plant resins, waxes, and other minor components), which are intended to provide a general descriptive framework rather than fixed or universal quantitative values [15].

Consistent with current understanding, propolis is more appropriately characterized on the basis of its botanical origin and associated chemical profiles, as the relative abundance of major constituent classes is known to vary substantially depending on plant sources, geographic location, and environmental factors [16]. Among them, Poplar-type propolis—abundant in Europe and North America—is derived primarily from *Populus* spp., particularly from *P. nigra*, which is rich in flavones, flavanones and cinnamic acid derivatives [6,7,17,18] (Figure S1). Other types, such as birch, green, red, Canary Islands and Pacific propolis, reflect the chemical signatures of their respective plant sources.

The close botanical and phytochemical relationship between *P. nigra* bud resin exudate and European propolis has been recognized for decades [4,19]. Seminal studies, including Greenaway et al. (1988) [20], demonstrated a striking congruence between poplar bud resinous exudate and propolis, with shared constituents such as galangin, pinocembrin and chrysin [19]. This evidence supports the classification of European propolis as Poplar-type propolis [4].

Because hive-collected propolis is subject to botanical and geographical variability as well as to hive contaminants [5,21], the direct extraction of *P. nigra* buds offers advantages in terms of standardization and functional reproducibility [19], making it particularly suitable for applications in pharmaceuticals, cosmetics, and nutraceuticals [18], and in the development of substance-based medical devices [22].

Here we report advanced targeted and untargeted analyses, including metabolomic fingerprinting, to provide molecular-level evidence of the compositional similarity between *P. nigra* bud and Poplar-type propolis extracts, where the shared features arise from the constituents of the bud resinous fraction, whereas the compositional divergences derive from metabolites associated with the bud tissues. Building on this comparative characterization, the study further investigates the applied potential of the patented *P. nigra* bud hydroalcoholic extract (PBHE) [23] by evaluating its performance within a finished oral spray formulation. This case study assessment focuses on three key bioactivities of therapeutic relevance—mucoadhesive capacity, barrier-forming properties and antibiofilm activity—using specific tests. Taken together, these functional insights aim to determine whether the *P. nigra* bud, including its resinous exudate, and corresponding extract PBHE can act as a standardized, bioactive and bee-independent alternative to traditional propolis, reinforcing its potential value within the modern use of natural substances for health, mucosal formulation science and industrial sustainable production.

## 2. Results and Discussion

A series of poplar bud and propolis samples were commercially sourced and analyzed using metabolomic techniques in order to elucidate their compositional features. Mass-spectrometry-based metabolomic approaches, including both untargeted and targeted strategies, are widely recognized as powerful tools for the comprehensive characterization of complex natural matrices, due to their high sensitivity, broad metabolite coverage, and capability to resolve subtle chemical differences [24,25]. Accordingly, these approaches were applied in the present study.

The marked compositional similarity observed between European propolis samples and *Populus nigra* buds provided a strong scientific rationale for the development of a novel extract (PBHE), obtained through a newly designed production process specifically aimed at concentrating *P. nigra* bud constituents. This process was subsequently patented [23]. The implementation of this unique and tailored procedure yielded an extract that, once used in the manufacture of formulated products, confers improved stability during downstream product manufacturing.

Within this framework, the results of the compositional characterization studies of *P. nigra* buds and European propolis extracts are presented and discussed, together with selected biological activities of the novel PBHE and of a PBHE-based formulated product developed as an oral spray.

### 2.1. Untargeted Metabolomic Fingerprint

A comparative untargeted metabolomic fingerprint analysis was conducted to evaluate the molecular similarity between propolis and bud extracts from *P. nigra*, which is widely recognized as the main botanical source of European propolis. To contextualize this relationship within a broader compositional framework, additional reference samples were included, encompassing botanically and biogeographically distinct matrices.

The investigated sample set (Table S1) included ten *P. nigra* bud samples, including eight dried samples and two freshly harvested buds, allowing the assessment of potential variability associated with post-harvest processing while maintaining a consistent botanical origin. In addition, one dried *P. balsamifera* bud sample was analyzed as a reference *Populus* species characterized by a distinct resin composition.

To further extend the compositional framework, six propolis samples of different botanical backgrounds were included, of which two were of Brazilian origin. Overall, this diversified yet focused sample set supported a comparative untargeted metabolomic approach aimed at assessing molecular similarity and compositional variability among *Populus*-derived bud exudates and propolis.

**Figure 2:**
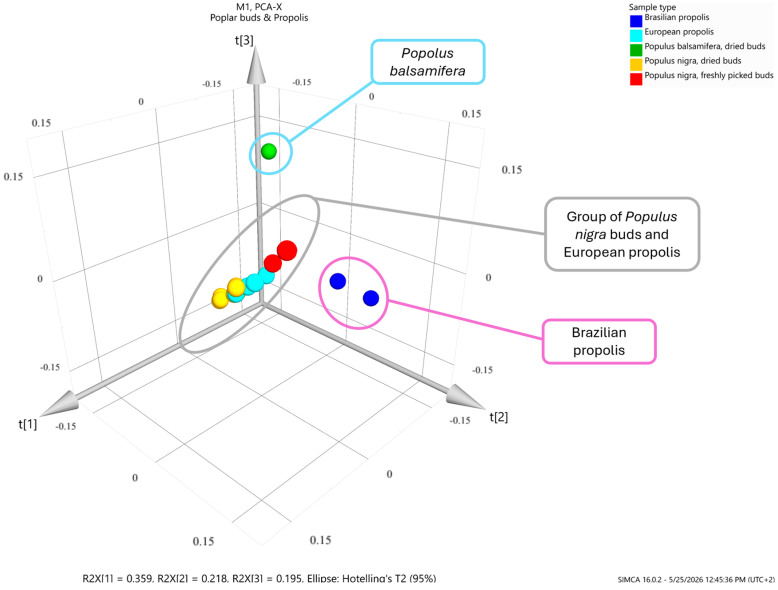
Untargeted metabolomics of different poplar buds and propolis.

*P. nigra* buds and propolis were analytically extracted with ethanol of high alcoholic strength (ethanol 75%), to efficiently solubilize the resinous fraction in agreement with real-world extraction processes [1,10,26], and the extracts were subjected to compositional comparison by an ESI-MS-based untargeted metabolomic fingerprint approach. Through principal component analysis (PCA) a statistical model was built (Figure 2) which revealed a distinct clustering of European propolis samples with *P. nigra* bud extracts. The samples formed a coherent group, clearly separated from Brazilian propolis and *P. balsamifera*. The most significant discriminative power occurred along the first two principal components (t1 = 35.9% and t2 = 21.8% of the variance), while information along the third component (t3 = 19.5% of the variance) also contributed meaningful statistical information. The observed separation pattern confirms that the propolis samples share a highly similar metabolomic signature with *P. nigra* bud exudate, while exhibiting marked compositional differences with other poplar species or non-poplar propolis types. Although the inclusion of additional *Populus* species and hybrids would further refine botanical discrimination, the present findings support *P. nigra* as the main botanical source underlying the metabolomic profile of European propolis.

### 2.2. Targeted Study

The compositional convergence between European propolis and *Populus nigra* buds observed in the untargeted study supported the rationale underlying the development of a novel hydroalcoholic extract (PBHE) [23].

Building on this evidence, a comprehensive phytochemical characterization was subsequently performed to elucidate the molecular composition of PBHE, to assess the effective enrichment of bud constituents, and to compare its compositional profile with that of a European propolis hydroalcoholic extract (PHE). Accordingly, rather than focusing on the quantification of a limited number of individual marker compounds, a targeted comprehensive compositional study was employed to evaluate the structural complexity of the investigated matrices. Given the chemical heterogeneity of the extracts—encompassing compounds with a wide range of molecular weights, polarities and solubilities—a multi-platform analytical strategy was adopted, based on the following techniques:

- GC-MS (Gas Chromatography–Mass Spectrometry).

This technique was employed for the analysis of volatile and semi-volatile organic compounds, particularly low molecular weight terpenoids and phenolic constituents.

- ^31^P-NMR (Phosphorus-31 Nuclear Magnetic Resonance Spectroscopy).

^31^P-NMR was applied to detect and quantify tannins and lignins after derivatization with organophosphorus reagent. This technique contributed to the characterization of the extract’s macromolecular polyphenols, in accordance with established methodologies reported by Argyropoulos [27] and Melone [28,29].

- ICP-OES/ICP-MS (Inductively Coupled Plasma Optical Emission Spectroscopy/Mass Spectrometry).

These techniques were utilized for the elemental analysis of inorganic constituents, including trace metals.

Targeted quantitative data and corresponding cumulative values, obtained by summing individually identified and quantified compounds (Table 1), were integrated with non-targeted complementary data (Table 2), including gravimetric determinations and UV-Visible spectrophotometric analysis. This integrated analytical framework was specifically adopted to overcome the intrinsic limitations of targeted techniques, which are inherently constrained by the limited availability of certified reference standards and may therefore underestimate the overall chemical complexity of natural matrices.

The combined evaluation of targeted and non-targeted datasets enabled a robust comparative assessment of PBHE and PHE. Targeted analytical characterization showed that both PBHE and PHE exhibited a highly significant, albeit incomplete, overlapping flavonoid and phenolic profile (Table 1), including characteristic poplar-type molecular markers such as pinocembrin, galangin, pinobanksin, naringenin, kaempferol, and isorhamnetin. Spectrophotometric and gravimetric data (Table 2) further confirmed the shared polyphenolic richness of the two matrices. Taken together, these results indicate that PBHE largely reproduces the polyphenolic complexity of PHE. A strong continuity was also observed for hydroxycinnamic acids, phenolic acids and terpenoids (Table 1), reinforcing the conservation of both polar and semi-volatile fractions across the two matrices.

Similarly, elemental profiling showed comparable macro- and micro-element patterns, suggesting that botanical and environmental factors, rather than bee-mediated processing, determine the inorganic signature of these materials (Table 1).

Even if certain quantitative differences were observed—such as the amount of salicylates, lignins, and tannins in PBHE (Table 1) associated with higher gravimetrically determined resinous fraction in PHE (Table 2)—these variations occurred against the background of significant molecular similarity. Such differences are plausibly attributable to the direct vegetal origin of PBHE, which includes internal bud tissues, versus the predominantly resinous exudates of PHE selectively collected by bees in propolis.

Overall, the convergence of targeted quantitative compositional data provides a coherent and compelling narrative: PBHE and PHE share a deep chemical kinship, rooted in their common origin from *P. nigra* bud resinous exudate. PBHE not only reproduces the defining phenolic–flavonoid–terpenoid framework of resinous European propolis but also captures a broader range of bud-derived constituents, characteristics of vegetal tissue rather than resinous exudate.

Therefore, these findings identify *P. nigra* buds as a botanical analogue of European propolis and pose PBHE as an analogue of European propolis extracts (such as PBE), exhibiting a highly similar chemical profile and therefore a comparable biological activity. This close chemical resemblance offers strong support for the functional interchangeability of PBHE and PHE in frameworks where a consistent, plant-based, and bee-independent source of poplar-type natural complex is required.

### 2.3. PBHE Biological Functionality

To support the functional relevance of the PBHE, a set of biological assays was conducted. In particular, antioxidant activity was evaluated using the DPPH assay, to highlight the correlation with the phenolic and flavonoid composition of the extract. In parallel, a biofilm formation inhibition assay was employed to assess the ability of PBHE to interfere with *Streptococcus pyogenes* biofilm development at sub-inhibitory (sub-MIC) concentrations. Ethanol was selected as the solvent for the biological experiments because the tested sample is intrinsically an alcoholic extract.

#### 2.3.1. Radical Scavenger Activity

The antioxidant activity of the poplar bud hydroalcoholic extract (PBHE) was evaluated using the DPPH (2,2-diphenyl-1-picrylhydrazyl) radical scavenging assay. DPPH is a stable chromogenic radical that undergoes a colorimetric change upon reduction in the presence of antioxidant compounds, which can be quantitatively measured by spectrophotometry. This assay aims to demonstrate the chemical antioxidant activity of the tested samples, based on hydrogen atom transfer to a free radical, without involving pharmacological, immunological, or metabolic mechanisms when applied to biological substrates.

**Figure 3:**
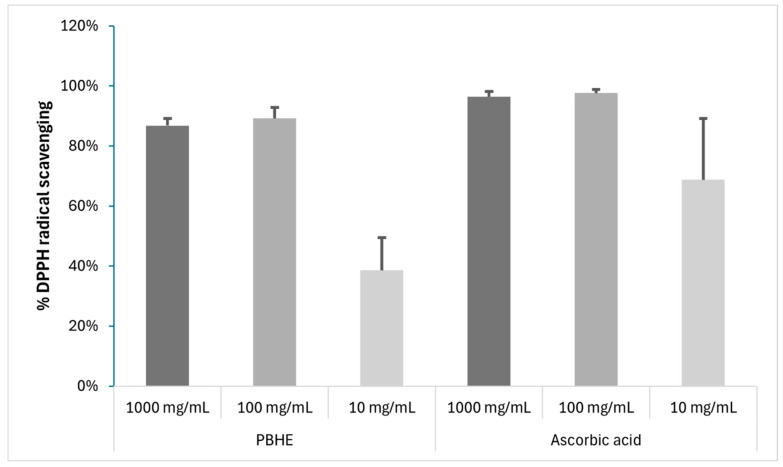
PBHE radical scavenging activity compared to that of vitamin C, used as positive control (the results are expressed as the mean of three independent analytical replicates).

The poplar bud extract was tested at concentrations of 1000, 100, and 10 μg/mL, selected to reflect concentrations relevant to its potential use in corresponding products. Vitamin C was used as a positive control. As shown in Figure 3, the poplar bud extract exhibited strong radical scavenging activity at 1000 and 100 μg/mL and moderate activity at 10 μg/mL, indicating a concentration-dependent antioxidant effect within the tested range. Importantly, the observed concentration dependence was assessed under conditions that are compatible with practical application, rather than across an extended concentration range aimed at pharmacological profiling. This approach allows a more realistic evaluation of the antioxidant potential of the extract in contexts relevant to its intended use.

#### 2.3.2. Inhibition of Biofilm Formation

The methodology employed in this assay leverages the ability of microorganisms to adhere to polystyrene surfaces and form biofilms, which were subsequently quantified by crystal violet staining. The bacterial strain selected was *Streptococcus pyogenes* (ATCC12344), a pathogen commonly associated with infections of the pharyngeal mucosa. The tested sample consisted of the PBHE prepared according to the previously described method and characteristics.

The assay was conducted in two phases. First, the minimum inhibitory concentration (MIC), defined as the lowest concentration of the extract capable of inhibiting bacterial growth, was determined. Gentamicin served as the positive control.

In the second phase, the capacity of the PBHE to inhibit biofilm formation was evaluated. Test samples were added to the culture medium at concentrations equal to or lower than the MIC to exclude the possibility that antibiofilm activity was attributable to growth inhibition.

Following an appropriate incubation period, the biofilms were stained with crystal violet. Excess dye was removed by washing with water, and the bound dye was solubilized in ethanol. The resulting solution was analyzed spectrophotometrically at 570 nm. The absorbance values were directly proportional to the biofilm biomass; therefore, antibiofilm activity was inversely correlated with absorbance. For each sample, the concentrations tested were 1× MIC, 0.5× MIC, and 0.1× MIC. The MIC corresponds to 0.22 mg/mL. Gentamicin was used at a concentration of 2 µg/mL. The data represent the mean of nine measurements obtained from three independent experiments performed in triplicate. Statistical significance was assessed using a *t*-test (** *p* < 0.001 for treated versus untreated bacteria).

**Figure 4:**
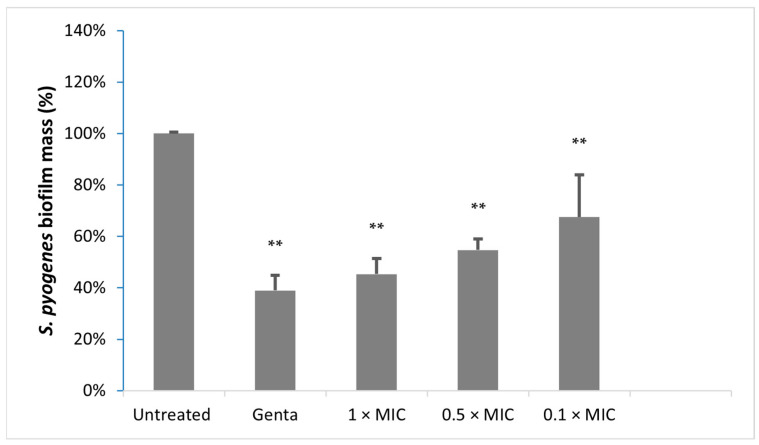
PBHE biofilm biomass formation inhibition assay (** *p* < 0.001). MIC corresponds to 0.22 mg/mL.

The results indicate that PBHE exhibited a good antibiofilm activity (Figure 4). Since this inhibition occurred at concentrations two- and ten-fold below the previously determined MIC, the observed effect is unlikely to be attributable to the direct antimicrobial activity of the extract.

An IC_50_ value of approximately 0.072 mg/mL has been previously reported for a poplar bud extract [30]. Although direct comparison should be interpreted with caution due to the different biological endpoints and experimental systems involved, the MIC value obtained for PBHE falls within the same order of magnitude. Differences may be partly explained by the distinct nature of the tested materials, as the extract in [30] is a dry extract, whereas PBHE is a liquid hydroalcoholic extract.

### 2.4. Specific Case—Biological Functionality of an Oral Spray Formulation (PBHE-SF) Containing Poplar Bud Hydroalcoholic Extract (PBHE)

Beyond the characterization of the PBHE itself, the biological functions of an oral spray formulation containing PBHE (PBHE-SF) were also investigated. From a regulatory perspective, for therapeutic products classified as medical devices [22,31], it is essential that biological functions should be demonstrated at the level of the final formulation rather than solely at the level of the active ingredient. Accordingly, PBHE-SF was designed, developed and studied as a representative technological application of PBHE within a medical device-type product intended for oropharyngeal protection, enabling evaluation of its functional performance within a realistic technological matrix.

The following section describes the biological properties of PBHE-SF, whose main component is PBHE formulated with glycerin, water, and natural flavouring agents (fluid composition 3, as defined in patent EP3463409 [23]).

The unique association of resinous and lignin fractions within the extract confers functional activities particularly suited for managing common disorders affecting the oropharyngeal mucosa. Accordingly, the experimental studies on PBHE-SF were designed to demonstrate three key mechanistic functionalities:

1. Mucoadhesive capacity, ensuring prolonged contact with the mucosal surface;
2. Barrier effect, providing protective action against irritant stimuli;
3. Ability to counteract bacterial biofilm formation, thereby contributing to oral cavity health.

All the biological assays were performed using the final formulated product as such or after dilution, and the experimental outcomes are reported with reference to the formulation itself rather than to the isolated PBHE. Ethanol was selected as the solvent for the biological experiments because the tested samples are intrinsically alcoholic formulations. The use of ethanol therefore ensured maximal compatibility between the vehicle and the samples, minimizing potential solvent-induced artifacts and reducing variability in data interpretation.

At the low final concentrations employed in the assays, ethanol is well documented to be biologically tolerated, while allowing an appropriate solubilization of the tested materials. Importantly, using ethanol instead of DMSO avoided introducing an additional solvent system not representative of the sample matrix, thereby providing a more realistic and conservative assessment of the biological effects of the tested extracts and product.

#### 2.4.1. Evaluation of Mucoadhesion Using a Cell-Based Assay

To exert its protective effect on the mucosal surface, a prerequisite is the PBHE-SF ability to adhere to the mucosa and remain in place for an adequate period of time. For this purpose, mucoadhesive capacity was investigated using in vitro cellular models following a validated experimental protocol. Specifically, the model was designed to assess adhesion to human buccal epithelial cells at different product dilutions and to evaluate resistance to simulated washing with a synthetic saliva flow.

The mucoadhesion test was performed according to a well-established experimental model [32,33], which accounts for the specific interactions involved in mucoadhesion.

A good mucoadhesive effect is an important requirement so that the product may remain in the site of action (pharyngeal mucosa) and mechanically carry out its protective actions.

The mucoadhesion of PBHE-SF was determined by an assessment of the product’s ability to adhere to cells by inhibiting lectin (a protein with a high affinity for glucoside and mannoside residues) bonding with membrane glycoproteins. The highest tested concentration corresponds to the undiluted product, which was included as the starting condition of the mucoadhesion assay. The extent of mucoadhesion was measured by a colorimetric reaction enabling us to quantify the sites on glycoproteins not engaged by the lectin, as engaged by the mucoadhesive product. The decrease in the absorbency value was proportional to the ability of the product to adhere (“mucoadhesion”) to the cells. The mucoadhesive ability is expressed as a percentage of inhibition of glycoprotein/lectin bonding. The observed, concentration-dependent reduction in lectin binding is consistent with a surface interaction mechanism between PBHE-SF and buccal epithelial cell membrane glycoproteins, as the colorimetric readout requires structurally intact cells for reproducible signal detection [32,33].

The confirmed data reported in Table 3 show how, above all at high dilutions (1:5), PBHE-SF has a good mucoadhesive effect.

As is evident from the above-reported data for the formulation dilute 1:2 (*v*/*v*), the spray based on poplar buds (PBHE-SF) has a good mucoadhesive ability under artificial salivary flow, up to two hours after the start of the test.

#### 2.4.2. Evaluation of Mucoadhesion Using an Inclined Plane Assay

- Methodology for Assessing Flavonoid Component Mucoadhesion Using a Pharyngeal-Cell-Coated Inclined Plane

At this stage, it is important to note that the mucoadhesive properties of PBHE-SF can be reasonably ascribed to the resinous component of the extract; however, it is equally essential that antioxidant compounds—such as flavonoids—remain adhered to the mucosal surfaces of the oropharyngeal cavity. To address this aspect, an additional cellular model based on human pharyngeal epithelial cells arranged on an inclined plane was developed. In this model, after allowing PBHE-SF to flow across the cell-coated surface, the absorbance of the fraction remaining adhered was quantified by UV-Visible spectrophotometry.

**Figure 5:**
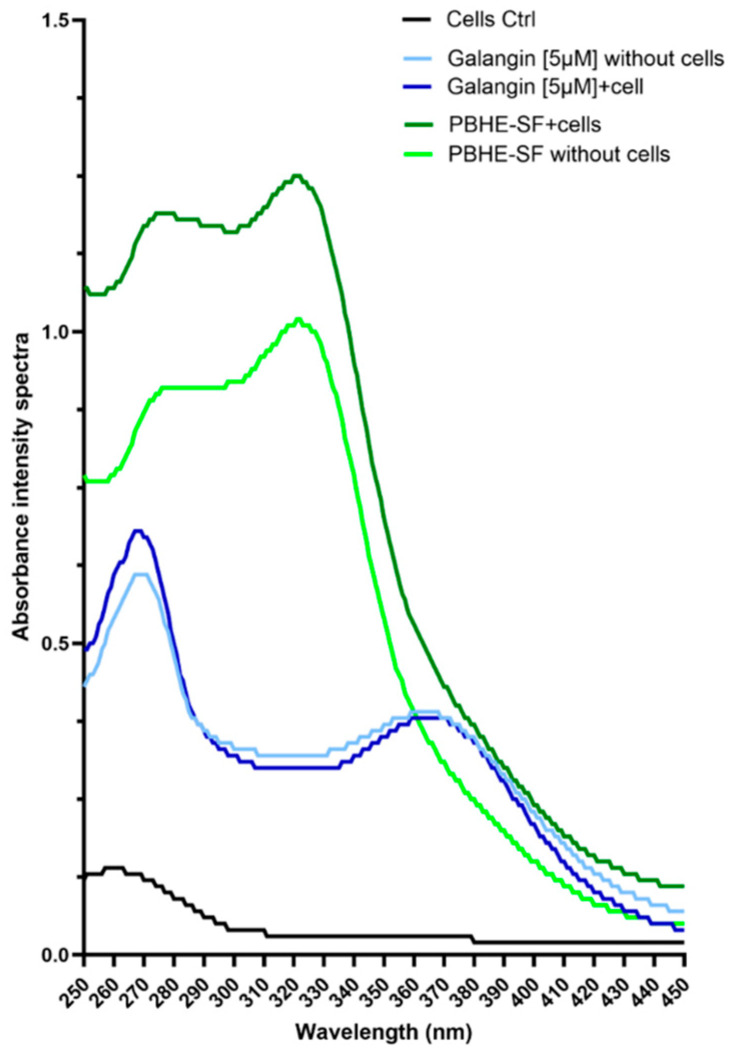
PBHE-SF adhesion to pharyngeal-cell-coated inclined plane, tested as such (undiluted). Light green line: Absorbance of the PBHE-SF molecular complex adhered to the surface in the absence of cells. Dark green line: Absorbance of the PBHE-SF molecular complex adhered to cells. Light Blue line: Absorbance of the flavonoid representative, galangin (pure standard), adhered to the surface in the absence of cells. Blue line: Absorbance of the flavonoid representative, galangin (pure standard), adhered to cells. Black line: Absorbance of the cells adhered to the surface (control).

The results, shown in Figure 5, reveal the UV-Vis spectra of PBHE-SF and galangin solution retained on the cellular monolayer compared with those obtained on the plastic substrate [34]. These findings clearly indicate that among the compounds retained on the mucosal surface—along with lignins and resinous matter—flavonoids and other polyphenolic constituents are also unequivocally present and may contribute to the observed functional bioadhesive properties.

- Methodology for Assessing Mucoadhesion Using a Mucin-Coated Inclined Plane

**Figure 6:**
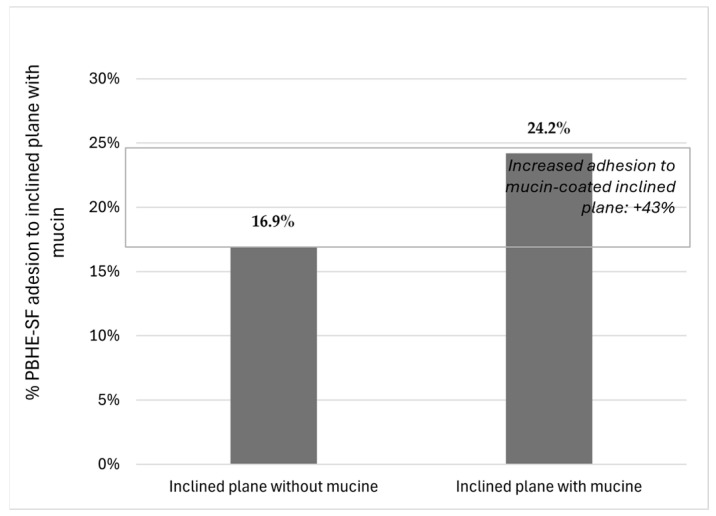
PBHE-SF adhesion to mucin-coated inclined plane.

Finally, Figure 6 illustrates that PBHE-SF exhibits a clear ability to interact with mucin when evaluated using the inclined plane assay. In this model, the inclined surface is tested either coated or uncoated with mucin [35]. The product is allowed to flow downward along both surfaces. As shown in the graph, PBHE-SF remains adhered to the mucin-coated plane 43% more than to the uncoated surface. This finding provides additional evidence of a physicochemical interaction between the formulation and the mucopolysaccharide, showing 43% higher retention on mucin than on the uncoated surface, a data consistent with the physicochemical interactions expected from the presence of the resinous-rich composition of PBHE (Table 3).

#### 2.4.3. Evaluation of Barrier Assay

The aim of the assay is to elucidate the mechanism of action of the PBHE-SF composition, by assaying its film-genic and protective ability compared to a known irritating agent; the agent used is lipopolysaccharide (LPS) membrane, a classic model of inflammatory induction. The assay [36] aims at highlighting the effective ability of the product to limit contact between the mucous membrane and external irritating agents.

The selected irritating agent was isolated from *Escherichia coli* cell membrane. The LPS dimensions are such as to allow consideration of the barrier’s effectiveness in protecting the mucous membrane from dust, smog, pollens and other agents concurring to irritation, and therefore to the onset of different pathologies burdening this particular and delicate environment.

Analyzing the experimental protocol in more detail, the sample’s ability to act as a barrier is assessed by measuring IL-6 production, which is a consequence of the contact between a layer of cells (fibroblasts) and the LPS. The experimental system provides the use of special Transwell wells, equipped with a collagen-coated semipermeable membrane that prevents direct contact between cells, deposited on the bottom, and the sample, stratified on the overhanging semipermeable membrane, which represents the sole communication route between the two portions of the well. LPS is inoculated in the space above the sample, whereby membrane crossing will be all the more difficult the greater the barrier effect exerted by the sample itself. Therefore, the quantification of interleukins produced at +24 h from LPS addition yields direct evidence of the barrier effect exerted by the assayed sample. IL-6 inhibition is a direct measurement of the barrier effect.

The barrier effect (BE) is expressed as a percentage of the reduction in the release of IL-6 and is obtained through comparison with the value obtained from the positive control (C+), i.e., from cells treated solely with LPS in the absence of the sample.

The experiment was conducted in triplicate and the results are reported in Table 4. Cytotoxicity was evaluated by assessing cell viability using the MTT colorimetric assay performed on the same cell cultures employed for the barrier test and internal control, allowing the exclusion of treatment- or procedure-related cytotoxic effects (Table S3). The cell-secreted IL-6 picograms after LPS insult and the % inhibition are compared to the positive control. The assay includes a positive control (C+), in which cells are exposed to LPS in the absence of any barrier sample, and a negative control (C−), in which cells are treated with culture medium alone. The magnitude of the inflammatory response is assessed by quantifying the cytokines released into the culture medium of the lower chamber at 24 h, with particular attention to interleukin 6 (IL-6), a marker characteristic of the late phase of the inflammatory cascade. IL-6 is not constitutively produced by human keratinocytes or fibroblasts but is synthesized by these cells in response to early pro-inflammatory signals such as IL-1α and TNF α, which are considered primary cytokines of the inflammatory response [37]. Detection of IL-6 following exposure to an irritant therefore provides a clear and specific indication of combined gene activation involving both early mediators and late phase IL-6, making it a reliable marker of effective cell-irritant contact [38].

**Figure 7:**
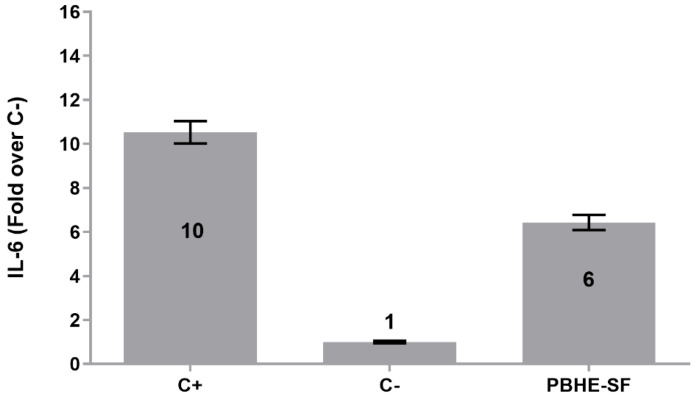
IL-6 production in the barrier assay. The graphs show the protection exerted by PBHE-SF on the cells and the data are expressed in terms of fold over (F.O.) compared to the C− control; fold over C− = [measured IL-6]/[IL-6 C−].

In Figure 7, data are reported in terms of fold over with respect to the negative control (C−).

From the mean IL-6 values measured in the barrier assay and in the positive control, the percentage of IL-6 release reduction is calculated. Therefore, applying PBHE-SF obtains a value of reduction in IL-6 production, which is of 39% compared to the positive control (Table 5).

**Figure 8:**
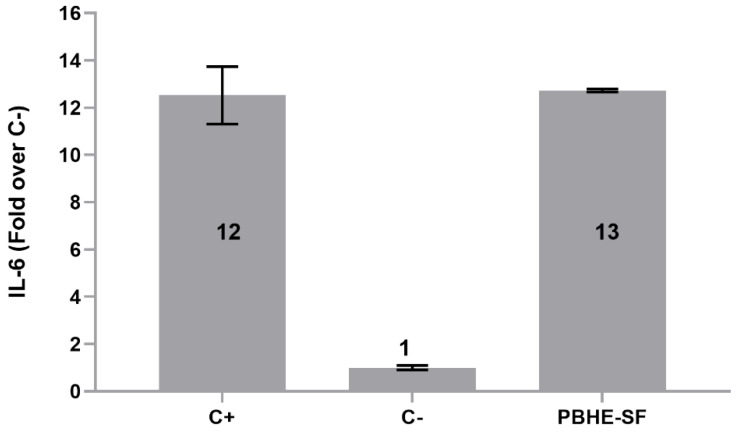
IL-6 production levels in cells treated with PBHE-SF, expressed as fold increase (fold over) relative to the internal control (C−). The PBHE concentration in PBHE-SF is 433.7 mg/mL. PBHE-SF contains 30% (*v*/*v*) ethanol, which was allowed to evaporate on the Transwell^®^ membrane prior to the start of the barrier test.

To validate the method used, and to verify that the results depend on the sole barrier effect of the assayed samples and exclude any pharmacological, immunological or metabolic effects (e.g., cytokine synthesis modulation), the barrier assay was assisted by an internal control (IC) concomitantly carried out on each sample. In the IC assay, first the cells are stimulated with the LPS, whereas the sample is added to the Transwell only subsequently: in this case, 0% inhibition of IL-6 would clearly indicate that the presence of the assayed composition affords a mechanical protection, in no way interfering with cellular cytokine synthesis. IL-6 concentration values measured in internal control experiments and where the treatment with PBHE-SF is carried out after LPS insult are reported in Table 6, while in Figure 8, the data are reported in terms of fold over with respect to the control (C−).

In the barrier assay pre-LPS challenge (Table 6), the formulation reduced LPS-induced IL-6 release by approximately 39% (Table 5), whereas in the internal control, where the product was added post-LPS challenge (Table 4), no inhibition was observed.

These data support a primarily mechanical barrier effect rather than a pharmacological modulation of cytokine synthesis.

#### 2.4.4. Evaluation of Bacterial Biofilm Dispersion

**Figure 9:**
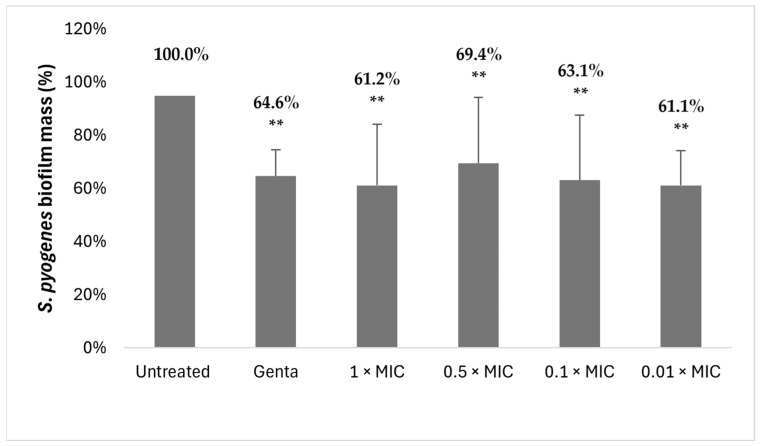
Quantification of *S. pyogenes* biofilm dispersion by PBHE-SF. The reported data represent the mean of nine measurements of crystal-violet-stained biofilm biomass, obtained from three independent experiments performed in triplicate. Statistical significance was assessed using a *t*-test (** *p* < 0.005; treated vs. untreated bacteria). The MIC corresponds to 2.13 mg/mL.

The activity of PBHE-SF in dispersing pre-formed biofilms was evaluated for *S. pyogenes*. PBHE-SF reduced the biomass of established biofilms at all tested concentrations, down to one-twentieth of the previously determined MIC (Figure 9). The MIC corresponds to 2.13 mg/mL. Gentamicin was used at a concentration of 2 µg/mL. These results demonstrated significant activity of PBHE-SF against pre-formed *S. pyogenes* biofilms. Under these conditions, the effects cannot be attributed to inhibition of microbial growth but rather indicate a direct impact on the established biofilm structure.

## 3. Materials and Methods

Samples. Botanical materials obtained from commercial suppliers were studied, including eight *P. nigra* bud samples, two freshly harvested *P. nigra* bud samples, one dried *P. balsamifera* bud sample, and six samples of propolis, of which one was a sample of black Brazilian propolis and one a sample of green Brazilian propolis (see Table S1).

The extracts Propolgemma^®^ (PBHE) and PHE were produced by Aboca S.p.A. (Sansepolcro, Italy), under proprietary processes. Propolgemma^®^ (PBHE)—at 62.7° alcohol degree—and the spray formulation (Propolgemma^®^ spray forte, PBHE-SF) were prepared in accordance with the procedures described in the corresponding patent [23]. Propolis hydroalcoholic multifraction extract (PHE) was at 70° alcohol degree. The PBHE-SF ingredients were vegetable glycerin, PBHE, water, natural citrus flavour, and essential oils of sweet orange and lemon, with an alcohol content of 30% *v*/*v*. The concentration of PBHE in PBHE-SF is 433.662 mg/mL [23].

Reagents and materials. Ethanol 75% *v*/*v* was prepared using analytical grade ethanol 96,4% and ultrapure water prepared in a Purelab Ultra water purification system (ELGA, Lane End, UK). Methanol LC-MS grade, isopropanol LC-MS grade, ESI Tuning Mix, sulfadimethoxine-D6, pyridine anhydrous analytical grade, N,O-bis(trimethylsilyl)trifluoroacetamide (BSTFA) with 1% trimethylchlorosilane (TMCS) ≥99%, deuterated chloroform NMR grade, 2-chloro-4,4,5,5-tetramethyl-1,3,2-dioxaphospholane ≥98%, chromium(III) acetylacetonate analytical grade, acetone analytical grade, dioxane anhydrous analytical grade, hydrochloric acid 0.1 M analytical grade, ethyl acetate analytical grade, hexane GC grade, sodium sulphate anhydrous analytical grade, nitric acid 70% *w*/*w* trace-metal grade and ammonium hydroxide 30% *w*/*w* trace-metal grade were purchased from Sigma-Aldrich (Milan, Italy).

The following analytical standards (see also Table S2) used in the targeted studies were purchased from Sigma-Aldrich (Merck KGaA) (Milan, Italy) or from Extrasynthese (Genay, France), depending on availability: 2-hydroxybenzyl alcohol, 3,4-dimethoxycinnamic acid, 3-salicylaldehyde, 4′,5-dihydroxy-7-methoxyflavone, 4-coumaric acid, 4-hydroxybenzoic acid, 4-methoxycinnamaldehyde, 4-methoxycinnamic acid, acetophenone, alloaromadendrene, caffeic acid, caffeic acid phenethyl ester, cantharidin, cedrol, chrysin, cinnamic acid, cinnamyl alcohol, citric acid, cholesterol, cyclohexanol, ferulic acid, galangin, gentisic acid, guaiol, isorhamnetin, kaempferol, methyl cinnamate, methyl salicylate, naringenin, nerolidol, oleic acid, pinobanksin, pinocembrin, pinostrobin, protocatechuic acid, salicin, salicylaldehyde, salicylic acid, sulfadimethoxine-D6, valencene, vanillic acid, α-bisabolol, α-curcumene, α-humulene and β-eudesmol. Certified multi-element ICP standards were purchased from Merck (Milan, Italy) or Inorganic Ventures (Christiansburg, VA, USA) depending on availability.

DPPH (2,2-diphenyl-1-picrylhydrazyl) analytical grade, vitamin C (ascorbic acid) analytical standard, Mueller–Hinton Broth (MHB), gentamicin pharma grade, crystal violet 4% microbiology grade, physiological saline (0.9% NaCl sterile), tris-buffered saline (TBS, 0.05 M, pH 7.6), trypan blue 0.5%, concanavalin A (biotinylated Con-A), streptavidin-peroxidase, o-phenylenediamine dihydrochloride (OPD), hydrogen peroxide, citrate-phosphate buffer 0.05 M, sulphuric acid 1 M, phosphate buffer pH 7, and mucin (porcine gastric mucin, analytical grade) were purchased from Fisher Scientific Italia (Milan, Italy). *Streptococcus pyogenes* (ATCC 12344^TM^) and human pharyngeal epithelial cells (FaDu (ATCC HTB-43) were obtained from the American Type Culture Collection (ATCC, Manassas, VA, USA).

Analytical measurements outsourced. The analytical measurements at Section 3.2.10, Section 3.2.11, Section 3.2.12 and Section 3.2.13 were outsourced to laboratory Neotron SpA (Modena, Italy), while those at Section 3.2.14 were outsourced to laboratory Mérieux NutriSciences Italy (Resana, Italy). The biological assay at Section 3.5 was outsourced to the laboratory of Francesco Paolo Bonina Professor Emeritus at the Department of Pharmaceutical Sciences, University of Catania, Viale A. Doria 6, 95125 Catania, Italy.

### 3.1. Untargeted Metabolomics Analysis

Sample Preparation. A total of 0.5 g of the sample (previously ground) was weighed into a centrifuge tube. To facilitate grinding, the sample was placed in a freezer for 5 min, then ground and weighed. The sample was extracted for 30 min with 30 mL of 75% *v*/*v* ethanol using an ultrasonic bath maintained at 35 °C. The extract was centrifuged at 4000 rpm for 5 min and decanted into a 100 mL volumetric flask.

A second extraction was performed on the residue under the same conditions; the extract was centrifuged and decanted into the same 100 mL flask. A third extraction was repeated on the residue under the same conditions and the extract was transferred into the same 100 mL flask. The sample was brought to volume with the extraction solvent after cooling the sample to 20 °C. The solution was filtered through a 0.45 µm cellulose acetate filter, then the filtrate was diluted 1:100 with the extraction solvent.

Analytical Conditions. Analyses were performed by FIA (Flow Injection Analysis) using an Agilent 1100 HPLC system (Agilent Technologies, Santa Clara, CA, USA) equipped with a thermostated autosampler and an Agilent SL ion trap mass spectrometer with an electrospray ionization (ESI) source (Agilent Technologies, Santa Clara, CA, USA).

FIA Parameters. The FIA analysis was performed by injecting a volume of 5 µL at a constant flow rate of 0.2 mL/min. The mobile phase consisted of ultrapure water (A) and methanol (B; LC-MS purity grade, 99.9%), mixed in an A/B ratio of 50:50. Each acquisition lasted 5 min. Between successive injections, a 15 min wash with a 50:50 H_2_O:MeOH mixture was carried out, after which the ion source was manually cleaned using a 50:50 isopropanol:H_2_O solution.

Calibration of the mass spectrometer was performed at the beginning of each analytical run using the calibrant suggested by the supplier (ESI Tuning Mix, Supleco, Bellefonte, PA, USA; Sigma-Aldrich, Milan, Italy; cod. 00036).

ESI (Negative Mode) Parameters. In negative ionization mode, the electrospray source was operated with a capillary voltage of +3000 V and an end-plate offset of −500 V. Nebulization was achieved at 40.00 psi, while the drying gas flow was maintained at 8.00 L/min with a drying temperature of 325 °C. Ion transfer conditions included a skimmer voltage of −33.1 V and a capillary exit voltage of −111.5 V. The ion optics were set as follows: Oct 1 DC −12.00 V, Oct 2 DC −1.70 V, and Oct RF 300.0 Vpp. Lens voltages were adjusted to 5.5 V for Lens 1 and 57.2 V for Lens 2. The trap drive was set to 45.2 V to optimize ion trapping and stability during mass analysis.

Multivariate statistical analysis. The spectra were processed using Data Analysis software version 2.2, converting them into matrices composed of three columns reporting the identified ions (*m*/*z* ratio), the absolute abundance, and the relative abundance, calculated by assigning the most intense peak a value of 100% and scaling all other signals accordingly. The resulting Excel file containing the original matrix was then exported as a TXT text file. All TXT files from all samples were aligned using SpecAlign software (Version 2.4, by Jason W.H. Wong), with BIN SIZE = 1, enabling the parameter “view as bar graph”. The processed datasets were saved in CSV format, and normalization of each signal was performed using the total sum method. Since the analysis was carried out in triplicate, the mean value of each triplicate series was also calculated.

The normalized matrix was subsequently imported into SIMCA-P+ (Umetrics, Umeå, Sweden; Version 13.0.3.0). Statistical tests Q^2^ and R^2^ were performed, yielding values of 0.187 and 0.772, respectively, indicating a robust model. Hotelling’s T^2^ and DModX parameters were evaluated, showing no evidence of strong or moderate outliers. Principal component analysis (PCA) was conducted by selecting the 3D Scores function. The PCA model was built using the first three principal components, providing a 3D representation at the 95% confidence level. The first three components accounted for 77% of the total model variance (PC1: 35.9%, PC2: 21.8%, PC3: 19.5%).

### 3.2. Targeted Analyses

A comprehensive characterization of *Populus nigra* (black poplar) bud hydroalcoholic extract PBHE and European propolis hydroalcoholic extract (PHE) was performed. A series of methods were developed and used to characterize extracts, identify compounds and screen their concentration in the samples.

#### 3.2.1. Determination of Phenols Using GC-MS Triple Quadrupole (TQ)

The PBHE and PHE were freeze dried, then the corresponding solid was used for analysis. Ten milligrams of sample were dissolved in 100 µL of pyridine, followed by the addition of 300 µL of BSTFA containing 1% trimethylchlorosilane and 0.25 mg of oleic acid (internal standard). The mixture was placed under an inert nitrogen atmosphere and heated to 90 °C for 1.3 h, then cooled to 20 °C, and 2.0 µL of the reaction mixture was injected into the GC-MS instrument (Varian 450 GC 320 MS, Agilent COMBO, Santa Clara, CA, USA). Analytical conditions: SLB™-5ms capillary column (30 m × 0.25 mm × 0.25 µm film thickness; Supelco, Bellefonte, PA, USA; catalogue no. 28471-U); helium carrier gas at 1.0 mL/min; oven program: 140 °C for 2 min, ramp at 5 °C/min to 300 °C, hold for 5 min; injector temperature 250 °C. Each analysis was performed in triplicate. Compound identification was carried out following two approaches:

(1) Comparison of mass fragmentation spectra with standard NIST libraries (NIST c4h10_ci, ch4_all, ch4_ci, c3oh_ci, ch4_drug, ch4_fda, aafs, libr_gp, libr_tr, libr_tx, demo, nh3_ci, NIST 21-LB, NIST107.LB);
(2) Comparison with authentic standards via spiking (standard addition method). Quantification was performed using the internal standard method (oleic acid) and applying the appropriate correction factors for each analyte.

Quantitative analysis was performed using the internal standard method, applying compound-specific conversion factors. Analytical standards (Table S2) were silylated under the same experimental conditions as the samples, and the internal standard (oleic acid) was added directly to the silylation reaction mixture. Conversion factors were calculated from the ratio between the chromatographic peak areas and the known molar amounts of each analyte relative to the internal standard. These conversion factors were subsequently applied for the quantitative determination of the analytes in each chromatogram.

#### 3.2.2. Determination of Lignin Using ^31^P-NMR Analysis [27]

The dried sample (1 g) was extracted using a 150 mL Soxhlet apparatus with 200 mL of acetone for 24 h under magnetic stirring and silicone oil bath heating. After extraction, the solid was transferred to a 100 mL Pyrex flask containing 45 mL dioxane + 5.0 mL 0.1 M HCl (9:1) and refluxed for 2 h under nitrogen. The mixture was vacuum-filtered first through a Büchner funnel (Whatman No.1), then through a Büchner funnel lined with celite. The filtrate was concentrated to ~10% of the original volume, mixed with 15 mL deionized water, and reconcentrated to remove excess dioxane. The aqueous phase was transferred to a 35 mL flask, acidified to pH 2.0–2.5 with HCl, frozen, thawed, and centrifuged (6000 rpm, 20 min). The pellet was washed with 0.05 M HCl, centrifuged, and the process repeated three times. The final pellet was lyophilized and stored under vacuum in a desiccator.

Thirty milligrams of lyophilized lignin were dissolved in 0.5 mL pyridine/CDCl_3_ (1.6:1.0). Then 0.1 mL each of freshly prepared 2-chloro-4,4,5,5-tetramethyl-1,3,2-dioxaphospholane (Cl TMDP) (phosphitylating reagent), chromium(III) acetylacetonate (5 mg/mL, relaxation reagent) and cholesterol (0.1 M, internal standard) were added. The solution was stirred for 2 h at room temperature and transferred to the NMR tube. Spectra were recorded on a 400 MHz ^31^P-NMR spectrometer (Bruker, Billerica, MA, USA), with a spectral window of 133–148 ppm.

The quantitative determination of lignins was performed by quantitative ^31^P-NMR after in situ phosphitylation of all labile H groups (aliphatic/phenolic OH and carboxylic acids) with Cl TMDP in anhydrous pyridine/CDCl_3_, using phosphitylated cholesterol as internal standard (with Cr(III) acetylacetonate as relaxation agent) and inverse gated decoupling; the integrated signal areas within the diagnostic chemical shift regions were converted into mmol/g of the corresponding functional groups (and total phenolics), providing a quantitative tannin fingerprint.

#### 3.2.3. Determination of Tannin Using ^31^P-NMR Analysis [28,29]

The dried sample (10 g) was suspended in 50 mL of 70% acetone in a 100 mL Pyrex flask and sonicated for 6 h. After centrifugation (18,000 rpm, 20 min), the supernatant was dried under reduced pressure; the aqueous phase was lyophilized and stored under vacuum. The lyophilized residue was dissolved in 80% methanol and chromatographed on a column packed with 4 g of Sephadex LH-20, eluting with 80% methanol. Fractions were monitored at 280 nm until absorbance < 0.05. The column was then eluted with 50% acetone, monitored at 400 nm until absorbance < 0.05. Fractions with absorbance > 0.05 were pooled, dried under reduced pressure, lyophilized, and stored under vacuum.

Thirty milligrams of lyophilized tannins were dissolved in 0.5 mL pyridine/CDCl_3_ (1.6:1.0). Then 0.1 mL each of 2-chloro-4,4,5,5-tetramethyl-1,3,2-dioxaphospholane, chromium(III) acetylacetonate (5 mg/mL) and cholesterol (0.1 M) were added. The mixture was stirred for 2 h at room temperature and transferred to the NMR tube. ^31^P-NMR spectra were acquired on a 400 MHz spectrometer (Bruker, Billerica, MA, USA), scanning from 133 to 148 ppm.

The quantitative determination of tannins was performed by quantitative ^31^P-NMR after in situ phosphitylation of all labile H groups (aliphatic/phenolic OH and carboxylic acids) with Cl-TMDP in anhydrous pyridine/CDCl_3_, using phosphitylated cholesterol as internal standard (with Cr(III) acetylacetonate as relaxation agent) and inverse gated decoupling; the integrated signal areas within the diagnostic chemical shift regions were converted into mmol/g of the corresponding functional groups (and total phenolics), providing a quantitative tannin fingerprint.

#### 3.2.4. Determination of Pinocembrin, Galangin by HPLC

The sample was thermostated at 20 °C and subsequently diluted with ethanol having the same alcoholic strength as the original hydroalcoholic extract, with a dilution factor of 1:10. The resulting solution was filtered through a 0.45 μm cellulose acetate membrane filter and then analyzed with an Agilent 1100 HPLC 1100 system (Agilent Technologies, Santa Clara, CA, USA).

Pinocembrin and galangin were used as analytical standards. Methanol (HPLC grade) was employed as the solvent for dissolution and dilution, and working standard solutions were prepared in the concentration range of 0.025–0.1 mg/mL. Chromatographic separation was performed on a reversed-phase column (Prodigy ODS3, 250 × 4.6 mm, 5 μm; Phenomenex, Torrance, CA, USA) equipped with a C18 security-guard cartridge (4 × 3 mm, 5 μm). The column temperature was maintained at 20 °C. Detection was carried out using a UV-Visible diode-array detector at 220 nm. The injection volume was 20 μL, and the mobile phase consisted of ultrapure water containing 0.2% (*v*/*v*) phosphoric acid (solvent A) and acetonitrile (solvent B). The chromatographic separation was performed using a gradient elution program starting with 70% solvent A and 30% solvent B at a flow rate of 1.0 mL min^−1^. After 43 min, the composition was adjusted to 60% A and 40% B while maintaining the same flow rate. At 43.5 min, the solvent composition remained unchanged, whereas the flow rate was reduced to 0.8 mL min^−1^ and maintained until 60 min.

At 62 min, the mobile phase composition was changed to 20% A and 80% B, and the flow rate was increased to 1.0 mL min^−1^. These conditions were held until 67 min. The system was then returned to the initial conditions (70% A and 30% B) at 72 min, with a flow rate of 1.0 mL min^−1^, allowing column re-equilibration. The total run time was 72 min, followed by a post-run equilibration time of 10 min. Quantification of pinocembrin and galangin in the samples was performed by external standard calibration. Calibration curves were constructed by plotting the chromatographic peak areas of the reference standards against their corresponding concentrations. The concentration of each analyte in the samples was determined by interpolation of the sample peak areas on the respective calibration curves. For each analyte, the calibration curve generated from peak area versus concentration data yielded a correlation coefficient *r* greater than 0.9. The linear working range was 0.1–0.025 mg/mL.

The percentage content of each compound in the analyzed samples was then calculated according to Equation (1):

Pinocembrin or galangin (mg/100 mL) = (A_c_ × C_s_ × F) × 100/A_s_ (1)

In the equation used for quantification, A_c_ represents the chromatographic peak area measured in the sample, A_s_ corresponds to the peak area of the reference standard, C_s_ indicates the concentration of the standard expressed in mg/mL, and F is the dilution factor applied to the sample prior to analysis.

Although the calibration curve was constructed using multiple concentration levels and verified for linearity, Equation (1) represents a simplified operational expression applied using the calibration point whose response was closest to that of the sample.

#### 3.2.5. Determination Caffeic Acid Phenethyl Ester (CAPE) by HPLC

The extraction and sample pre-treatment procedures were carried out as described in Section 3.1, employing the same instrumentation.

CAPE was used as the analytical standard. Methanol (HPLC grade) 90% (*v*/*v*) was employed as the solvent for dissolution and dilution, and working standard solutions were prepared in the concentration range of 0.0125–0.05 mg/mL. Chromatographic separation was performed on a reversed-phase column (Prodigy ODS3, 250 × 4.6 mm, 5 μm; Phenomenex, Torrance, CA, USA) equipped with a C18 security-guard cartridge (4 × 3 mm, 5 μm). The column temperature was maintained at 60 °C. Detection was carried out using a UV-Visible diode-array detector at 220 nm. The injection volume was 20 μL, and the mobile phase consisted of ultrapure water containing 0.2% (*v*/*v*) phosphoric acid (solvent A) and acetonitrile (solvent B). The flow rate was set at 1.0 mL min^−1^. The chromatographic separation was performed using a gradient elution program starting with 70% solvent A and 30% solvent B. After 43 min, the composition was adjusted to 60% A and 40% B. At 45 min, the solvent composition changed to 20% solvent A and 80% solvent B. These conditions were held until 50 min. The system was then returned to the initial conditions (70% A and 30% B) at 55 min, allowing column re-equilibration. The total run time was 55 min, followed by a post-run equilibration time of 5 min. Quantification of CAPE in the samples was performed by external standard calibration. Calibration curves were constructed by plotting the chromatographic peak areas of the reference standards against their corresponding concentrations. The concentration of each analyte in the samples was determined by interpolation of the sample peak areas on the respective calibration curves. The calibration curve generated from peak area versus concentration data yielded a correlation coefficient *r* greater than 0.9. The linear working range was 0.05–0.0125 mg/mL.

The percentage content of CAPE in the analyzed samples was then calculated according to Equation (2):

CAPE (mg/100 mL) = (A_c_ × C_s_ × F) × 100/A_s_ (2)

The parameters in the quantification equation are as previously defined in Section 3.2.4. Equation (2), derived from a calibration curve established over multiple concentration levels and verified for linearity, was applied as a simplified operational expression using the calibration point with a response closest to that of the sample.

#### 3.2.6. Determination of Salicin by UPLC-qToF

The extract was filtered through a 0.2 μm membrane filter. An aliquot of 1 mL was transferred into an autosampler vial and spiked with 20 μL of sulfadimethoxine-D6 (approximately 0.005 mg/mL), used as the internal standard. The vial was vortex-mixed and subsequently injected into the UPLC-QToF-MS system (Xevo G2XS System; Waters, Milford, MA, USA). Chromatographic separation was performed on a Cortecs C18 column (2.1 × 100 mm, 1.6 μm, Waters, Milford, MA, USA) equipped with a VanGuard pre-column (2.1 × 5 mm, Waters, Milford, MA, USA), with the column temperature maintained at 30 °C. The mobile phase consisted of water and methanol, both containing 0.1% (*v*/*v*) formic acid, delivered at a constant flow rate of 0.3 mL min^−1^. Separation was achieved using a gradient elution program starting with 99% aqueous phase and progressively increasing the organic phase, followed by a high-organic wash and subsequent re-equilibration to the initial conditions.

Mass spectrometric detection was carried out using a time-of-flight analyzer equipped with an electrospray ionization source operated in negative ion mode. Source and desolvation parameters were optimized according to the tuning indication of the LC-MS producers. Data were acquired in MS^e^ mode over the time window from 3.8 to 6.5 min, using a mass range of *m*/*z* 50–1200. Low-energy collision was disabled, whereas high-energy collision was applied in the range of 12–25 V to enable fragmentation. The analyzer was operated in sensitivity mode, with a scan time of 0.1 s and data collected in continuum format. Data acquisition was performed using MassLynx v.4.1 software, while the acquired data was processed using TargetLynx application manager integrated in MassLynx v.4.1 (Waters, Milford, MA, USA). For qualitative analysis, the analyte was identified in the samples by comparison of its retention time and accurate mass with those of the corresponding reference standard. For quantitative analysis, salicin was quantified using the low-energy acquisition function by constructing an external calibration curve, with signal normalization to the internal standard. The resulting calibration curve exhibited good linearity over the investigated concentration range, with a coefficient of correlation *r* greater than 0.99.

#### 3.2.7. Determination of Total Flavonoid Content Expressed as Galangin by Spectrophotometric Method

The sample was diluted 1:500 with ethanol having the same alcoholic strength as the original hydroalcoholic extract at 20 °C. The total flavonoid content, expressed as galangin, was calculated according to Equation (3), taking into account that the specific absorbance (A1%, 1 cm) of galangin at 353 nm is equal to 600.67:

Total flavonoids as galangin (mg/100 mL) = [(A × F)/600.67] × 10 × 100(3)

In the equation, A refers to the absorbance of the sample measured at 353 nm, while F represents the dilution factor applied to the sample.

Although modern AlCl_3_-based assays are reported to allow the quantitative determination of specific flavonoid subclasses, this internal method supported by consolidated historical data was considered more appropriate for the purposes of the present study.

#### 3.2.8. Determination of Terpenes Using GC-MS Single Quadrupole (Q)

PBHE (10 mL) and PHE (5 mL), were processed separately by transferring the appropriate volume into a Falcon tube and mixing with 25 mL of hexane. The mixture was extracted by vortex mixing for 10 min and subsequently centrifuged at 4000 rpm for 5 min. The organic supernatant was carefully transferred into a previously tared 50 mL volumetric flask using a glass Pasteur pipette. The extraction was repeated once under identical conditions, and the combined organic phases were brought to 50 mL with hexane. A small amount of anhydrous sodium sulphate was added to remove residual moisture, and the solution was filtered through a 0.45 μm syringe filter. An aliquot of 180 μL of the filtered extract was transferred into a micro vial and spiked with 20 μL of cyclohexanol internal standard (22.6 ppm). The sample was then injected into the GC-MS system.

Gas chromatographic analysis was performed using Agilent 6890N Network GC System (Agilent Technologies, Santa Clara, CA, USA) equipped with a SPLIT SPLITLESS injector and coupled to a 5973 mass selective detector, with a single quadrupole analyzer. Separation was achieved on a VF-5MS fused-silica capillary column (J&W Scientific, Agilent Technologies, Santa Clara, CA, USA); a chemically bonded stationary phase composed of 5% phenylmethylpolysiloxane and 95% dimethylpolysiloxane (30 m × 0.25 mm i.d., 0.25 μm film thickness) was used for the analysis.

The injector temperature was set at 300 °C. Mass spectrometric detection was carried out in SCAN mode. Helium was used as the carrier gas at a constant flow rate of 1.0 mL min^−1^. The injection volume was 1 μL, using splitless injection mode. The solvent delay was set to 3.50 min.

The oven temperature program was as follows: initial temperature of 40 °C, held for 1 min; ramped at 30 °C min^−1^ to 100 °C with no hold; increased at 5 °C min^−1^ to 190 °C with no hold; then ramped at 35 °C min^−1^ to 300 °C and held for 5.86 min. The total run time was 30 min. The quantitative determination was carried out by correlating the areas of the sample with the areas of the reference standards, using a calibration curve, and normalization on the internal standard.

#### 3.2.9. Determination of Resinous Matter

An aliquot of the thermostated extract (20 mL for PBHE or 40 mL for PHE), maintained at 20 °C, was evaporated to dryness under reduced pressure using a rotary evaporator in a 100 mL round-bottom flask. The resulting resinous residue adhering to the flask walls was recovered by rinsing with 50 mL of ethyl acetate. As the residue was not completely soluble, ultrasonic treatment was applied to facilitate detachment from the flask walls. The entire recovered suspension was transferred into a 50 mL Falcon tube and extracted by vortex mixing for 2 min. The Falcon tube was centrifuged for 5 min at room temperature, and the supernatant was transferred into a previously tared 100 mL round-bottom flask. The flask tare (TP) was determined by drying the flask in a ventilated oven at 105 ± 2 °C for 2 h, cooling in a desiccator to room temperature, and recording the weight. The ethyl acetate extract was evaporated to dryness under reduced pressure in the tared flask. A second extraction was performed on the residue from the first extraction using an additional 50 mL of ethyl acetate. This solvent volume was also used to rinse the first evaporation flask. The ethyl acetate was then transferred to the same Falcon tube used for the first extraction and processed under identical vortexing and centrifugation conditions. After centrifugation, the supernatant was combined with the residue from the first extraction in the same tared flask and evaporated to dryness under reduced pressure. The flask containing the final residue was placed in an oven at 102 °C for 2 h, then cooled in a desiccator for at least 1 h and finally weighed.

The total resinous matter content was calculated according to Equation (4):

Total resinous matter (mg/mL) = [(P_l_ − T_p_)/V_i_] × 1000 (4)

In the equation, P*l* corresponds to the final gross weight of the flask containing the dried residue, expressed in milligrams; T*p* represents the tare weight of the empty flask, also expressed in milligrams; and V*i* indicates the initial volume of extract, expressed in millilitres.

#### 3.2.10. Determination of Total Fats

The determination was carried out using a gravimetric method, in accordance with the procedure described in ISTISAN Report 1996/34 [39]. The method is based on the isolation of the analyte by solvent extraction, followed by evaporation of the solvent and gravimetric determination of the dried residue.

#### 3.2.11. Determination of Total Proteins

Total nitrogen content was determined using the Kjeldahl method, in accordance with AOAC Method 920.70 [40]. Briefly, the sample was subjected to acid digestion to convert organic nitrogen into ammonium sulphate, followed by alkaline distillation and titrimetric determination of the released ammonia. The protein content was subsequently calculated by converting the measured total nitrogen content using an appropriate nitrogen-to-protein conversion factor. Unless otherwise specified, a conversion factor of 6.25 was applied, assuming an average nitrogen content of 16% in proteins. Protein content was therefore expressed as total nitrogen × 6.25.

#### 3.2.12. Determination of the Ash Content

The ash content was determined using a gravimetric method, in accordance with the procedure described in ISTISAN Report 1996/34 [41]. An aliquot of the sample was accurately weighed into a previously tared porcelain or platinum crucible and incinerated in a muffle furnace at 550 °C until complete combustion of the organic matter and attainment of constant mass. The ash content was calculated gravimetrically from the weight of the inorganic residue remaining after incineration and expressed as g per 100 g of sample.

#### 3.2.13. Determination of Elements

ICP-MS method. An aliquot of the sample was accurately weighed (2 g) and spiked with a known amount of an internal standard. Digestion was carried out on a heating block, with 15 mL of concentrated nitric acid (HNO_3_, 65–70% *w*/*w*) in a digestion tube, keeping the solution boiling for 90 min. After digestion, the resulting cooled solution was brought to 50 mL with ultrapure water in the same digestion tube with water and filtered prior to ICP-MS analysis.

In order to assess the linearity of the analytical method, a series of standard additions was performed on a 10 mL aliquot of the sample solution. Four calibration levels were generated by spiking the sample solution with increasing concentrations of selected elements. Specifically, chromium and selenium were added at 25, 50, and 100 µg/L; whilst copper, manganese, iron, and zinc were fortified at 100, 200, and 400 µg/L. These fortified solutions, together with the unspiked sample, were subsequently analyzed, using an analytical blank as the reference solution.

For quantification purposes, the elements were further determined using the standard addition technique. To this end, two solutions were prepared: the unspiked sample solution and a second solution enriched with a single concentration level of each target analyte. Chromium and selenium were spiked at 100 µg/L, whereas copper, manganese, iron, and zinc were each added at 400 µg/L. Both the native sample and the spiked sample solutions were then analyzed under identical conditions, again employing an analytical blank as the reference. The method’s sensitivity was established through the determination of the limit of quantification (LQ) and the limit of detection (LD). For copper, iron, manganese, zinc, chromium, and selenium, the LQ was 0.005 mg/kg, whilst the LD was 0.0025 mg/kg for each element.

For iodine [42] the sample (5 g) was subjected to alkaline digestion using 30 mL of 30% (*w*/*w*) ammonium hydroxide (NH_4_OH) in a glass flask. The digestion was performed on a hot plate, keeping the solution boiling for 20 min. After cooling to room temperature, the digested solutions were quantitatively transferred to 250 mL volumetric flasks and brought to volume with ultrapure water. In parallel with the sample analysis, a reagent blank containing only 30% NH_4_OH and a certified reference material with a known iodine content were processed under the same conditions. The resulting solution was then filtered to remove any residual particulates and was subsequently analyzed by ICP-MS.

Four calibration levels were generated by spiking the sample solution with increasing concentrations of the element. Specifically, iodine was added at 25, 50, and 100 µg/L. These fortified solutions, together with the unspiked sample, were subsequently analyzed, using an analytical blank as the reference solution. The method’s sensitivity was established through the determination of the limit of quantification (LQ) and the limit of detection (LD), which were 0.005 mg/kg and 0.0025 mg/kg, respectively.

This dual approach—a linearity assessment followed by a standard addition quantification—ensured reliable calibration within the sample matrix and enabled the accurate determination of the selected trace elements. The performance characteristics demonstrate the method’s suitability for trace-level determination of metals in low-concentration matrices.

Instrumental determinations were performed by inductively coupled plasma mass spectrometry (ICP-MS) using an Agilent 7500CE ICP-MS system (Agilent Technologies, Santa Clara, CA, USA).

ICP-OES method. The analytical procedure was carried out in accordance with EPA Method 6010D [43]. An aliquot of sample (0.5 g) was digested with 65–70% (*w*/*w*) nitric acid at gentle boiling using a heating block for 90 min. After cooling at room temperature, the solution was brought to a final volume of 50 mL with ultrapure water in the same digestion tube. The resulting solution was then filtered to remove any residual particulates and was subsequently analyzed by inductively coupled plasma optical emission spectrometry (ICP-OES).

Calibration curves of sodium, potassium, calcium, magnesium, and phosphorus were constructed using a five-point external calibration ranging from 0 to 120 mg/L for all analytes. The emission wavelengths selected for the measurement of the elements were 589.59 nm for sodium, 766.49 nm for potassium, 422.67 nm for calcium, 285.21 nm for magnesium and 213.61 nm for phosphorus. Yttrium, measured at 371.03 nm, was employed as an internal standard to compensate for potential fluctuations in plasma stability and matrix-related signal suppression. All the measurements were conducted using an Optima 7300 DV spectrometer (PerkinElmer, Waltham, MA, USA) operated under standard multi-element conditions.

The linearity of the calibration model was evaluated by determining the correlation coefficient (*r*) for each analyte across the calibration range. An acceptance threshold of *r* > 0.995 was applied in accordance with typical validation criteria for quantitative spectroscopic methods, and all elements demonstrated correlation coefficients exceeding this limit, thereby confirming the suitability of the selected concentration interval for precise quantitative analysis.

Method sensitivity was assessed through the determination of the limits of detection (LD) and quantification (LQ) under the analytical conditions employed. For sodium and potassium, LQ and LD values were established at 1.0 mg/kg and 0.5 mg/kg, respectively. Calcium and magnesium exhibited lower detection thresholds, with LQ values of 0.4 mg/kg and LD values of 0.2 mg/kg. Phosphorus, owing to its higher background emission and more challenging spectral environment, showed an LQ of 4.0 mg/kg and an LD of 2.0 mg/kg.

These values collectively demonstrate that the analytical procedure possesses adequate sensitivity for the determination of these macro-elements in the analyzed matrices.

#### 3.2.14. Determination of Citric Acid

A total of 3 g of the sample was added into a 100 mL volumetric flask. The volume was made up with deionized water, and the solution was stirred on a magnetic stirrer for approximately 30 min. The resulting mixture was filtered through a 0.45 µm cellulose syringe filter. The filtered solution was then subjected to instrumental analysis with an Agilent 1100 HPLC system (Agilent Technologies, Santa Clara, CA, USA).

The chromatographic analysis was carried out using an Aminex HPX-87H column (300 × 7.8 mm, 9 µm; Bio-Rad, Hercules, CA, USA), with a 4 mM sulfuric acid solution as the mobile phase, delivered at a flow rate of 0.6 mL/min under isocratic conditions. The injection volume was 20 µL, and the total analysis time was 60 min. Detection was performed using a UV-Visible detector at a wavelength of 214 nm. Within each analytical batch, the following solutions were injected sequentially: a diluted reference solution containing citric acid at approximately 15 mg/L, a reference mixture containing citric acid within the concentrations range of 300–400 mg/L, and the solution obtained from the sample. Prior to injection, all the solutions were filtered through a 0.45 µm cellulose syringe filter. The limit of quantification (LoQ) of the method was 0.01%. Analyte identity was confirmed by comparing the UV absorption spectrum acquired across the chromatographic peak in the sample chromatogram with that obtained from the corresponding reference material. The method exhibited a linear response over the concentration range up to 400 mg/L. The calibration curve generated from peak area versus concentration data yielded a correlation coefficient *r* greater than 0.9.

The content of citric acid in the analyzed samples was calculated according to Equation (5):

Citric acid (mg/mL) = (Ac × Cs × F)/As(5)

As previously specified in Section 3.2.4, the parameters used in the quantification equation retain the same definitions.

### 3.3. DPPH Assay for Radical Scavenger Activity

The scavenger anti-oxidizing activity directed by the extract of poplar buds was assessed by a DPPH (2,2-diphenyl-1-picrylhydrazyl) assay. DPPH is a stable violet-coloured radical that undergoes a reduction to a yellow chromophore when it reacts with an antioxidant compound; this change can be quantified spectrophotometrically at 517 nm. The assay, therefore, measured the intrinsic chemical scavenging capacity of a sample based solely on hydrogen-atom transfer to a free radical, without the involvement of pharmacological, immunological or metabolic mechanisms. The sample, tested at concentrations of 1000, 100 and 10 µg/mL, was incubated with the DPPH ethanol solution for 30 min. Its scavenging activity was compared to that of vitamin C (ascorbic acid), used as the positive control. The percentage of DPPH radical quenching was calculated according to Equation (6):

% DPPH Radical Scavenging = [1 − (Abs_sample_ − Abs_blank_)/Abs_control_] × 100 (6)

Absorbance readings were corrected using an appropriate blank consisting of the sample diluted in ethanol. All the experiments were conducted in triplicate to ensure reproducibility.

### 3.4. Biofilm Formation Inhibition Assay

Bacterial biofilm formation by *Streptococcus pyogenes* was evaluated in 96-well polystyrene microtiter plates in the presence of different concentrations of the tested extract and product. The methodology used exploits the intrinsic ability of microorganisms to adhere to polystyrene surfaces and develop structured biofilms, which can then be quantified by crystal violet staining.

After determining the minimum inhibitory concentration (MIC) of the sample, the ability of the same to inhibit or reduce biofilm formation was assessed at concentrations corresponding to 1 × MIC, 0.5 × MIC and 0.1 × MIC for PBHE, and 1 × MIC, 0.5 × MIC, 0.1 × MIC, and 0.05 × MIC for PBHE-SF.

Microorganisms were incubated for 24 h in Mueller–Hinton Broth (MHB) supplemented with 2% sucrose, in the presence of the three different concentrations of the sample. Following incubation, planktonic cells were removed, and the adhered biofilm biomass formed at the bottom of the wells was quantified by crystal violet staining. Each well was washed with physiological saline and stained with 4% crystal violet. After staining, the biofilm layers were rinsed with water, and the retained dye was solubilized in ethanol to enable spectrophotometric quantification. The absorbance of the resulting solution was measured spectrophotometrically at 570 nm, providing a quantitative estimate of the biofilm mass.

The reported data are the mean of 9 measurements carried out in triplicate. Statistical significance was calculated with the *t*-test (treated bacteria versus untreated bacteria).

- MIC Evaluation

The Minimal Inhibitory Concentration (MIC) was determined by the microbroth dilution method according to the Clinical and Laboratory Standards Institute/National Committee for Clinical Laboratory Standards (CLSI/NCCLS) Approved Standard M100-S21, 2007). The microorganisms were grown in Mueller–Hinton Broth for 18 h at 37 °C. On the day of the assay, bacterial cells were harvested by centrifugation and adjusted to a final concentration of 10^5^ CFU/mL. The PBHE or PBHE-SF was maintained in stocks at a concentration of 100 mg/mL. The sample was then serially diluted 1:2 in 96-well U-bottom microtiter plates. Gentamicin 2 µg/mL was used as the positive control.

Following dilution of the sample, 100 µL of the microbial suspension was added to each well. The plates were incubated for 24 h at 37 °C. After incubation, bacterial growth in each well was visually assessed. The MIC was defined as the lowest dilution of the sample at which no visible microbial growth was detected.

### 3.5. Mucoadhesion Assay—Method of Adhesion to Cells

Materials. Human buccal epithelial cells were collected from the oral cavity of eight healthy donors. Streptavidin-peroxidase, biotinylated lectin (Con-A), o-phenylenediamine dihydrochloride (OPD), trypan blue, hydrogen peroxide, and all other reagents used in the study were purchased from Sigma Chemical Company (St. Louis, MO, USA).

Description. The model derives from the optimization of previous experimental protocols and scientific works on the topic [32,33,44,45]. Mucoadhesivity is determined by evaluation of the percentage of inhibition of the lectin-glycoprotein bond induced by the analyzed sample using buccal mucosal cells pretreated with the sample under examination. The cells were initially pretreated for 15 min with the sample and were subsequently treated with biotinylated lectin (Con-A), to which streptavidin peroxidase was subsequently added, making it possible to form the protein-glucose-lectin-biotin-streptavidin peroxidase complex. At this point, the cells were washed and the protein-glucose-lectin-biotin-streptavidin peroxidase complex was quantified, thanks to the presence of the peroxidase, by means of a reaction of oxidation of the ortho-phenylenediamine.

The protein/glucose/lectin/biotin/streptavidin peroxidase complex catalyzes the polymerization reaction:

O-phenylenediamine + H_2_O_2_ —(streptavidin-peroxidase catalyst)→ 2,3- diaminophenazine (yellow product).

The intensity of the yellow/orange coloration of the solution (measured using a spectrophotometer with = 450 nm) was proportional to the quantity of glycoprotein/lectin bonds and therefore to the quantity of available sites (glycoproteins) for mucoadhesion.

The absorbance value thus determined constituted the “control”. In the mucoadhesion assay on the buccal cells, the cells were treated for about 15 min at 30 °C with the sample before the treatment with lectin. In the presence of a mucoadhesive sample, that sample will inhibit lectin bonding, decreasing, proportionally to their mucoadhesion ability, the signal strength in the sample compared to the control as described above. The percentage of mucoadhesion of the sample is calculated according to Equation (7):

Mucoadhesion (%) = [1 − (Abs_sample_/Abs_control_)] × 100 (7)

In this calculation, Abs_sample_ is the absorbance of the cells pretreated with the mucoadhesive substance and the Abs_control_ is the absorbance of the untreated control cells subjected to lectin binding.

This mucoadhesion assay is designed to evaluate the functional interaction between the sample and buccal epithelial cell surface glycoproteins; therefore, it does not include a specific cell viability endpoint. The analytical readout relies on structurally intact cells, since disruption of the plasma membrane would result in the loss of organized glycoproteins and consequently in non-reproducible lectin binding.

Assessment of Mucoadhesion. The oral cavity of 13 healthy male and female donors (aged 21–42 years), who had abstained from food and drink for at least 60 min, was gently scraped using a sterile wooden spatula. The collected cells were immediately suspended in Tris-buffered saline (TBS; 0.05 M, pH 7.6). After counting with 0.5% trypan blue, the suspensions were diluted with 0.9% NaCl to obtain a final concentration of 480,000 cells per sample.

The cells were maintained at 4 °C until processing. The cell suspensions were centrifuged at 2000 rpm for 5 min and incubated with 5 mL of PBHE-SF, undiluted, diluted 1:2 or 1:5 in 0.9% NaCl, to a final volume of 5 mL. Control samples were incubated with 5 mL of 0.9% NaCl alone. Incubation was performed for 15 min at 30 °C under gentle agitation.

After three washes with TBS, the buccal cells were incubated with 5 mL of Con-A (10 mg/L) at 30 °C for 30 min, washed three times with TBS, and subsequently incubated for 60 min at 30 °C with 5 mL of streptavidin-peroxidase (5 mg/L).

Following an additional three washes, 240,000 cells per sample were transferred into 2.5 mL of OPD in citrate-phosphate buffer (0.05 M) containing H_2_O_2_. After 5 min, the reaction was stopped with 1 M H_2_SO_4_. Absorbance values for each determination were recorded by UV-Visible spectrophotometry.

Because PBHE-SF exhibits intrinsic coloration that could interfere with absorbance readings, a 0.9% NaCl solution was used as the reading reference, and an appropriately diluted solution of the sample in 0.9% NaCl was used as the blank. All the samples were analyzed in triplicate, and the results are reported as mean ± SEM.

- Evaluation of Mucoadhesive Retention Under Salivary Flow

A Franz diffusion cell system composed of six independent chambers was used to evaluate the retention of mucoadhesion over time. Franz cells are widely employed to study permeation processes through biological or artificial membranes [45]. Each cell comprises a donor compartment and a receptor compartment (Figure S2).

For this experiment, the donor compartments were loaded with buccal cell cultures prepared as described above and treated with the tested sample. Among the dilution ratios examined, the 1:2 dilution was selected for the retention study, as it was considered the most physiologically relevant under in vivo conditions.

The donor compartment was perfused with a continuous flow (2 mL/min) of artificial saliva, consisting of an isotonic aqueous phosphate buffer (pH 7) containing 0.5% mucin. A six-channel peristaltic pump delivered saliva simultaneously to all Franz cells. The artificial saliva solution was thermostated at 36 °C to mimic physiological temperature.

A cellulose acetate membrane was placed at the interface between the donor and receptor compartments, allowing the passage of saliva while retaining the mucosal cells in the donor chamber. The receptor compartment was initially filled with artificial saliva and thermostated at 36 °C via the Franz cell water jacket.

The experiment was conducted for 0.5, 1, and 2 h. At each time point, the cells from the donor compartment were collected and subjected again to biotinylated lectin (Con-A) binding, followed by streptavidin-peroxidase incubation and reaction with OPD, following the same methodology described for the initial mucoadhesion assay. This allowed the quantification of residual mucoadhesion after exposure to the salivary flow. Each experimental condition (each time point) was performed in triplicate.

### 3.6. Mucoadhesion Assay

#### 3.6.1. Method of Adhesion to Pharyngeal Cells on Inclined Plane 

A monolayer of FaDu human pharyngeal epithelial cells was cultured on standardized plastic substrates (Nunc Lab-Tek Flask on Slide; Thermo Scientific, Carlsbad, CA, USA) [35]. The sample PBHE-SF was applied (300 µL) to the FaDu (ATCC-HTB-43)-coated plane, which was subsequently inclined at 45°, and allowed to slide downward for 30 s at 37 °C. Galangin (282200; Merck) [46] was dissolved at the working concentration of 5 µM in sterile-filtered 1× phosphate-buffered saline (PBS; pH 7.4; Thermo Fisher Scientific) containing 30% (*v*/*v*) ethanol (Thermo Fisher Scientific), an alcoholic strength aligned with the composition of PBHE-SF. In an initial phase, the parallel adhesion of PBHE-SF and galangin solution [5 µM] was evaluated on both a cell monolayer and a cell-free plastic substrate, with the aim of distinguishing adhesion specifically mediated by biological interactions vs. a non-specific adhesion on plastic support.

The slides were then allowed to dry under a ventilated hood for 1 h. Subsequently, the cells, together with the fraction of product that remained adhered, were mechanically detached using a cell strainer and recovered in 3 mL of PBS containing 30% *v*/*v* of ethanol, corresponding to the same ethanol concentration present in PBHE-SF. The recovered solutions were then diluted threefold and dispensed into spectroscopic microplates (UV-Star™ 96-Well UV; Greiner Bio-One, Kremsmünster, Austria). The absorbance of the retained fraction was quantified by UV-Vis spectrophotometry from 250 to 450 nm.

#### 3.6.2. Mucoadhesion Assay—Method of Adhesion to Mucin on Inclined Plane

Measurements were carried out using an apparatus consisting of a 45° inclined plane thermostated at 37 °C, beneath which a microbalance was positioned. The microbalance recorded weight variations at 1 s intervals, printing the corresponding values onto paper. The acquired data were subsequently transcribed and processed using an Excel spreadsheet.

The inclined plane served as the support for the biological substrate, consisting of a mucin film. The mucin layer was prepared by depositing 3 mL of an 8% (*w*/*w*) suspension of porcine gastric mucin in phosphate buffer (pH 6.4) onto a horizontally positioned plexiglass plate. The dispersion was allowed to dry for 12 h at room temperature under a chemical fume hood, yielding a film with a defined surface area of 33.6 cm^2^.

The quantity of mucin, its concentration, and the deposition method were optimized during method development, and minor adjustments were implemented as required. Because a solvent such as high-grade ethanol can strongly affect the integrity of the mucin film, an exact amount of sample (20 mg) was accurately weighed. PBHE-SF was initially dissolved in 1 mL of ethanol (≤70% *v*/*v*) and subsequently diluted 1:2 in medium to obtain a final concentration of 10 mg/mL, thereby reducing the ethanol content in the test conditions. The sample was dispensed onto the measurement surface using a multi-channel pipette calibrated to deliver 250 µL at constant timing and speed over a 6 s interval. The total amount deposited per test was 1 mL (the exact mass was measured beforehand and depended on sample density).

The sample was applied to the upper region of the inclined mucin-coated plane and allowed to slide downward. The microbalance recorded, as a function of time, the amount of sample reaching it, corresponding to the portion not retained by the substrate. Each experiment lasted 40 s; however, no further changes in measured mass were observed after approximately 20 s.

Control measurements were also performed in the absence of the mucin film (blank tests), applying the same sample volume and experimental conditions but onto a clean inclined surface. This allowed assessment of the intrinsic flow behaviour of the sample independently of its interaction with the biological substrate.

For each sample the percentage of sample remaining attached either to the mucin film or to the inclined plane (blank) at the end of the measurement, normalized to the initial amount applied, was calculated. Then, the percentage difference between the amount adhered to the mucin film and that adhered to the blank surface was calculated using Equation (8):

Difference % = (% adhered to mucin − % adhered blank)/% adhered blank(8)

In the calculation, the % adhered to mucin is the percentage of sample retained on the mucin film at the end of the test and the % adhered blank is the percentage of sample retained on the inclined plane in blank measurements.

### 3.7. Barrier Assay

The model is based on the principle that cells exposed to an inflammatory stimulus produce and secrete pro-inflammatory mediators (cytokines) into the extracellular environment in amounts proportional to the degree of inflammation induced [47]. Within a defined range, a direct relationship exists between the concentration of the inflammatory agent and the quantity of cytokines released [47]. In this system, a primary human fibroblast line (HUDE) is employed [47]. The assay uses two chambers physically separated by a semipermeable membrane that permits the diffusion of sufficiently small solutes (Figure S3).

In the lower chamber, consisting of the well of a standard cell culture plate, fibroblasts are grown under controlled conditions. The upper chamber, corresponding to a Transwell^®^ insert, contains the inflammatory agent—lipopolysaccharide (LPS). A thin film of the test sample is applied to the surface of the semipermeable membrane that separates the two compartments, allowing assessment of its potential barrier effect (BE) in preventing or reducing the free passage of the inflammatory stimulus. Depending on the insulating capacity of the sample, the diffusion of LPS from the upper to the lower chamber is reduced, resulting in diminished stimulation of cytokine production by the fibroblasts [48,49].

To ensure that the results reflect solely the barrier effect of the tested samples—and not an anti-inflammatory interference with cytokine synthesis—an internal control (IC) is performed in parallel for each sample. In the IC procedure, the cells are first stimulated with LPS and only subsequently exposed to the sample [48,49]. This allows verification that the barrier applied to the Transwell^®^ insert has been appropriately prepared, as any reduction in cytokine synthesis in this configuration would indicate undesired direct pharmacological interaction rather than a mechanical isolation effect.

Cell Culture Model. Human fibroblasts (HuDe) were seeded in 24-well plates at a density of 40,000 cells per well in minimum essential medium (MEM) supplemented with 2 mM glutamine and 10% fetal bovine serum. The cells were incubated overnight at 37 °C in a humidified atmosphere enriched with 5% CO_2_ before assay preparation.

Preparation of Test Samples and Internal Controls. The sample was tested as is, without dilution. A volume of 60 µL of sample solution was layered on the inner surface of the Transwell^®^ membrane (Transwell, 6.5 mm 24-well, 0.4 μm, Fisher Scientific Italia, Milan, Italy) separating the two chambers in order to evaluate its barrier effect against the free passage of the inflammatory agent LPS (1 μg/mL), which was added to the bottom of the upper chamber for 1 h, as represented by the cell culture insert. Following deposition, the sample was allowed to adhere to the membrane under a biological safety cabinet. Under these conditions, ethanol acts solely as a formulation solvent and is expected to largely evaporate prior to cell exposure, leaving a residual film responsible for the barrier effect. The absence of ethanol or diffusible component-mediated anti-inflammatory effects was experimentally verified through the IC procedure and further confirmed by cell viability assessment.

Depending on the barrier-constituting capacities of the sample, a decrease in the migration of the inflammatory agent from the upper chamber will be observed, resulting in less stimulation of the cells towards the production of cytokines. The test also included a positive control in which the cells were treated with LPS in the absence of the tested sample, along with a negative control (untreated cells) in which the cells were treated with the culture medium alone, in the absence of the sample being tested. The extent of the inflammatory reaction was estimated after 24 h via a quantitative determination of IL-6 release in the lower chamber—a response typical of the late phase of the inflammatory reaction [50]. The IC involves the same steps as the BT, but with different dynamics, apt to highlight any inflammatory activity exerted via mediators putatively diffusing from the product barrier; the same quantity of LPS used in the BT was left free to diffuse from the upper to the lower chamber and, after just 1 h, 60 μL of the sample solution was layered on the inner surface of the Transwell membrane. In this set-up, the observation of anti-inflammatory activity could be ascribable to the diffusion of interactors from the barrier, rather than to the barrier effect itself. The quantification of IL-6 in supernatants was performed using ELISA (ab178013, Abcam, Cambridge, United Kingdom), according to the manufacturer’s instructions. Following the collection of the supernatants, cell viability was assessed via an MTT assay (Sigma-Aldrich, Milan, Italy), according to the manufacturer’s instructions.

Assessment of the Barrier Effect. The BE is expressed as the percentage reduction in IL-6 release compared with the positive control (C+). IL-6 quantification was performed using a sandwich ELISA on the supernatant collected 24 h after treatment, with cytokine concentration derived from the regression curve generated using known standard concentrations. The result is expressed according to Equation (9):

Barrier effect % = inhibition % of IL-6 release(9)

Cell viability assessment. Cell viability was assessed by the MTT colorimetric assay (Sigma Aldrich) in the same cell cultures subjected to barrier test and internal control treatments. This analysis was performed to exclude any cytotoxic effects related to either the experimental procedures or the applied treatments and to ensure that any lack of IL-6 production was not attributable to a reduction in viable cell numbers. Cytotoxicity was expressed as the percentage reduction in cell viability relative to untreated control cells, which were defined as exhibiting 0% viability reduction. Data were obtained from three independent experiments performed in triplicate.

The percentage inhibition of cell viability was calculated according to Equation (10):

Cell Viability Inhibition (%) = 100 − [(%VCT/%VCNT) × 100](10)

where %VCT represents the percentage viability of the treated cells (the cells exposed to the test sample, positive control, and negative control), and %VCNT represents the percentage viability of the cells not included in the test (the untreated control cells).

## 4. Conclusions

Consistent with the previous literature, it was confirmed through ESI-MS untargeted metabolomic fingerprinting that the exudate of *P. nigra* buds exhibits a compositional profile highly consistent with that of temperate European propolis.

This evidence further supports the development of a *P. nigra* bud hydroalcoholic extract (PBHE) [23] as a potentially equivalent, plant-based alternative to propolis, a natural material frequently affected by intrinsic compositional variability as well as by contamination arising from substances used within hive management.

The new PBHE was studied from a compositional standpoint using a targeted analytical approach. It was shown that it shares a highly conserved chemical profile with the alcoholic propolis extract PHE, including overlapping signatures of phenols, flavonoids, hydroxycinnamic acids, and terpenoids. The analytical evidence therefore classify PBHE within the same phytochemical family as Poplar-type propolis, while retaining additional bud-derived constituents—such as salicylates, lignins, and tannins—that are characteristic of bud plant tissue and largely absent from the resinous exudate, representing a coherent botanical analogue of temperate European propolis extract.

Beyond compositional similarity to PHE, PBHE demonstrated a functional profile consistent with the bioactivities historically attributed to propolis, such as concentration-dependent antioxidant activity [5] and the ability to inhibit biofilm formation [51]. Notably, for PBHE the antibiofilm activity was demonstrated at sub-MIC concentrations against *Streptococcus pyogenes*, underscoring the ability to disrupt early biofilm formation independently of bactericidal activity.

Importantly, the development of a spray formulation containing PBHE (PBHE-SF) [23], classified as a medical device for oropharyngeal application, confirms the effective translation of the extract’s intrinsic molecular complexity into formulation-relevant biological functions. These include strong mucoadhesive capacity under dilution and flow conditions, retention of antioxidant flavonoids on epithelial substrates, mechanical barrier formation as well as the ability to interfere with established *Streptococcus pyogenes* bacterial biofilms.

A major strength of this work is the integrated, multi-platform strategy that links metabolomic profiling with formulation-level functional testing. A few general considerations should nonetheless be noted. First, as is typical for complex natural matrices, some aspects of the chemical characterization remain influenced by methodological constraints, which future studies may refine further. Second, the functional assessment is based mainly on in vitro systems, useful for mechanistic interpretation but not fully representative of in vivo conditions. Additional studies expanding these aspects would naturally help to consolidate and extend the present findings.

Collectively, these findings substantiate PBHE as a reliable and standardizable plant-derived source of poplar bud-type molecular complexes, providing functional equivalence to European propolis without dependence on bee-mediated processing. Such independence from biological variability—particularly from forage patterns, seasonality and hive-specific conditions or treatment-related contaminants—confers PBHE a significant advantage in terms of manufacturing standardization, enabling controlled sourcing and batch-to-batch reproducibility. PBHE-based formulation therefore offers a scientifically substantiated alternative for medical device applications aimed at mucosal protection, providing a natural complex with consistent composition, documented performance, and a clear mechanistic basis for its barrier-forming, mucoadhesive and antibiofilm properties and representing a robust platform for scalable and sustainable industrial development.

## 5. Patents

The work reported in this manuscript is partially related to the patent EP 346 409B1 granted to Aboca S.p.A., of which Luisa Mattoli, Andrea Lugli and Anna Maidecchi are inventors.

## Figures and Tables

**Table 1:** Analysis of PBHE and PHE with targeted methods.

Method	Compounds	PBHE, mg/100 mL *	PHE, mg/100 mL *
	**Resinous compounds (Phenols and Terpenes)**	**246.8**	**204.6**
	**Phenols, total**	**228.9**	**186.4**
	**Flavonoids, total**	**159.6**	**166.8**
	**Flavones, total**	**8.6**	**2.3**
GC-MS (TQ)	4′,5-Dihydroxy-7-methoxyflavone	<LQ	0.07
GC-MS (TQ)	Chrysin	6.1	0.2
GC-MS (TQ)	Naringenin	2.5	2.0
	**Flavonols and Dihydroflavonols, total**	**76.8**	**110.6**
HPLC-UV	Galangin	61.4	89.5
GC-MS (TQ)	Isorhamnetin	0.6	0.5
GC-MS (TQ)	Kaempferol	11.2	18.3
GC-MS (TQ)	Pinobanksin	3.6	2.3
	**Flavanones, total**	**74.8**	**54.3**
HPLC-UV	Pinocembrin	73.4	53.1
GC-MS (TQ)	Pinostrobin	1.4	1.2
	**Salicylates, totals**	**24.7**	**1.6**
GC-MS (TQ)	3-Salicylaldehyde	0.1	0.1
GC-MS (TQ)	Methyl salicylate	0.1	<LQ
UHPLC-qToF	Salicin	23.9	1.0
GC-MS (TQ)	Salicylic acid	0.2	0.1
GC-MS (TQ)	Salicylaldehyde	0.4	0.4
	**Phenolic Acids, total**	**4.1**	**1.4**
GC-MS (TQ)	4-Hydroxybenzoic acid	0.4	1.2
GC-MS (TQ)	4-Coumaric acid	3.4	<LQ
GC-MS (TQ)	Gentisic acid	<LQ	0.0025
GC-MS (TQ)	Protocatechuic acid	0.2	0.2
GC-MS (TQ)	Vanillic acid	0.1	0.02
	**Hydroxycinnamic Acids, total**	**8.5**	**8.4**
GC-MS (TQ)	3,4-Dimethoxycinnamic acid	1.4	1.4
GC-MS (TQ)	4-Methoxycinnamic acid	0.8	0.6
GC-MS (TQ)	4-Methoxycinnamaldehyde	<LQ	1.3
GC-MS (TQ)	Caffeic acid	2.7	0.03
HPLC-UV	Caffeic acid phenethyl ester (CAPE)	1.8	1.2
GC-MS (TQ)	Cinnamic acid	0.4	0.6
GC-MS (TQ)	Ferulic acid	1.2	3.3
GC-MS (TQ)	Methyl cinnamate	0.2	<LQ
NMR	**Lignin, total**	**18.0**	**0.9**
	**Tannins, total**	**13.4**	**7.3**
NMR	Condensed Tannins	2.2	2.1
NMR	Hydrolysable tannins (Gallotannins)	7.7	4.2
NMR	Hydrolysable tannins (Ellagitannins)	3.5	1.0
	**Terpenes, total**	**12.1**	**14.9**
	**Sesquiterpenes, total**	**12.1**	**14.9**
GC-MS (Q)	α-Bisabolol	1.1	1.2
GC-MS (Q)	α-Curcumene	2.0	3.6
GC-MS (Q)	α-Humulene	0.3	0.7
GC-MS (Q)	β-Eudesmol	3.6	4.1
GC-MS (Q)	Alloaromadendrene	<LQ	0.3
GC-MS (Q)	Cedrol	<LQ	0.02
GC-MS (Q)	Guaiol	5.0	4.8
GC-MS (Q)	Nerolidol	<LQ	0.03
GC-MS (Q)	Valencene	<LQ	0.2
	**Other Aromatic Compounds, Total**	**5.8**	**3.3**
GC-MS (TQ)	2-Hydroxybenzyl alcohol	0.7	<LQ
GC-MS (Q)	Cinnamyl alcohol	5.1	3.1
GC-MS (Q)	Acetophenone	<LQ	0.1
GC-MS (Q)	Cantharidin	<LQ	0.1
	**Organic Acids, total**	**12.1**	**<LQ**
HPLC-UV	Citric acid	12.1	<LQ
	**Minerals**, **total**	**33.71**	**7.93**
	**Macro-Elements, total**	**33.4**	**7.6**
ICP-OES	Calcium	1.6	0.5
ICP-OES	Magnesium	6.3	0.4
ICP-OES	Phosphorous	2.6	0.3
ICP-OES	Potassium	19.1	1.9
ICP-OES	Sodium	3.8	4.5
	**Micro-Elements, total**	**0.31**	**0.33**
ICP-MS	Chromium	0.001	0.003
ICP-MS	Copper	0.02	0.01
ICP-MS	Iodine	0.004	0.003
ICP-MS	Iron	0.14	0.01
ICP-MS	Manganese	0.03	0.02
ICP-MS	Selenium	0.001	0.001
ICP-MS	Zinc	0.11	0.28

* The reported values represent the mean of determinations performed on two different production batches.

**Table 2:** Analysis of PBHE and PHE using non-targeted method.

Method	Compounds	PBHE, mg/100 mL *	PHE, mg/100 mL *
Gravimetric	Resinous matter, total	1655.0	2265.0
Spectrophotometric	Flavonoids total, expressed as galangin	559.6	526.5
Gravimetric	Minerals total, determined as ash	153.6	97.3
Gravimetric	Proteins, Total	<LQ	<LQ
Gravimetric	Fats, total	<LQ	<LQ

* The reported values represent the mean of determinations performed on two different production batches.

**Table 3:** Mucoadhesion and resistance to washing of PBHE-SF.

Test	Conditions (*)	PBHE-SF (**)
% Mucoadhesion	Undiluted formulation (as such)	76.3 ± 5.4
	Formulation diluted 1:2 (*v*/*v*)	66.4 ± 6.1
	Formulation diluted 1:5 (*v*/*v*)	54.6 ± 5.1
Resistance to washing	Formulation diluted 1:2 (*v*/*v*), 0 h	66.4 ± 6.1
	0.5 h	63.6 ± 5.3
	1 h	51.6 ± 4.8
	2 h	43.1 ± 4.4

(*) The formulated product (PBHE-SF) was tested as such (undiluted) and after dilution. Accordingly, the final ethanol concentrations were 30% (undiluted), 15% (1:2), and 6% (1:5), respectively. The corresponding PBHE concentrations were 433.7 mg/mL (undiluted), 216.8 mg/mL (1:2), and 86.7 mg/mL (1:5). (**) Statistical analysis. Mucoadhesion test: undiluted formulation vs. 1:2 dilution (*p* > 0.05); 1:2 vs. 1:5 dilution (*p* > 0.05); undiluted formulation vs. 1:5 dilution (*p* < 0.05). Resistance to washing: 0.5 h vs. 0 h (*p* > 0.05); 2 h vs. 1 h (*p* > 0.05); 1 h vs. 0.5 h (*p* < 0.05); 1 h vs. 0 h (*p* < 0.01).

**Table 4:** IL-6 production in the barrier assay where fibroblasts are treated with PBHE-SF.

Sample	IL-6 (pg/μL)	Mean	SD
PBHE-SF + LPS 1	392.915	413.188	22.109
PBHE-SF + LPS 2	409.888		
PBHE-SF + LPS 3	436.761		
Control + 1 (LPS)	639.728	676.973	32.634
Control + 2 (LPS)	690.646		
Control + 3 (LPS)	700.547		
Control − 1 (MEM)	61.945	64.303	3.489
Control − 2 (MEM)	62.652		
Control − 3 (MEM)	68.310		

Note: PBHE concentration in PBHE-SF is 433.7 mg/mL. PBHE-SF contains 30% (*v*/*v*) ethanol, which was allowed to evaporate on the Transwell^®^ membrane prior to the start of the barrier test.

**Table 5:** IL-6 release inhibition percentage with PBHE-SF.

Sample	IL-6 Release Inhibition %	Mean	SD
PBHE-SF + LPS 1	41.960	38.965	3.266
PBHE-SF + LPS 2	39.453		
PBHE-SF + LPS 3	35.483		

Note: PBHE concentration in PBHE-SF is 433.7 mg/mL. PBHE-SF contains 30% (*v*/*v*) ethanol, which was allowed to evaporate on the Transwell^®^ membrane prior to the start of the barrier test.

**Table 6:** IL-6 production in barrier assay with PBHE-SF (tested as such, undiluted) and controls.

Sample	IL-6 (pg/μL)	Mean	SD
PBHE-SF + LPS 1	839.865	839.158	4.637
PBHE-SF + LPS 2	843.401		
PBHE-SF + LPS 3	834.208		
Control + 1 (LPS)	914.828	825.721	80.223
Control + 2 (LPS)	803.091		
Control + 3 (LPS)	759.244		
Control − 1 (MEM)	72.553	65.953	6.378
Control − 2 (MEM)	59.874		
Control − 3 (MEM)	65.481		

Note: The PBHE concentration in PBHE-SF is 433.7 mg/mL. PBHE-SF contains 30% (*v*/*v*) ethanol, which was allowed to evaporate on the Transwell^®^ membrane prior to the start of the barrier test.

## Data Availability

The data supporting the findings of this study are made available from the corresponding author upon specific request.

## Supplementary Materials

The following supporting information can be downloaded at: https://www.mdpi.com/article/10.3390/molecules31111836/s1. Figure S1: Schematic representation of the major constituent classes commonly reported for propolis. This overview is intended as a general descriptive framework; the relative proportions of these components are known to vary widely depending on botanical and geographical origin. Figure S2: Franz diffusion cell used for assessing the resistance of the formulations’ mucoadhesive properties under artificial salivary flow. Figure S3: In vitro Transwell-based exposure model used to evaluate the barrier-forming capacity of the test samples. Table S1: Data of samples analyzed with untargeted approach. Table S2: Data of compounds analyzed with targeted methods. Table S3: Cell viability and cell viability inhibition assessed in the barrier test and internal control.
