# Supplementary material for: Populus nigra Bud Extract as a Standardized Alternative to Propolis: Evidence of Compositional Similarity—Functional Properties of an Oral Spray Containing Populus nigra Bud Extract
Source: Molecules. 2026 May 26;31(11):1836. doi: 10.3390/molecules31111836 (PMC13258445; doi:10.3390/molecules31111836)
Supplement: Supplementary file 1 [file molecules-31-01836-s001.zip › molecules-4254995-supplementary.pdf]

SUPPLEMENTARY INFORMATION

Populus nigra Bud Extract as a Standardized Alternative to Propolis: Evidence of Compositional Similarity—Functional Properties of an Oral Spray Containing *Populus nigra* Bud Extract

Luisa Mattoli [1,*,†], Andrea Lugli [1,†], Michela Burico [1], Giada Fodaroni [1], Denise Decarli [1], Mattia Gianni [1], Anna Maidecchi [1], Giulia Antonini [1], Silvia Tondi [1], Anna Gaetano [1], Valentina Fiordelli [1], Rita Pagiotti [2], Jacopo Lucci [2], Claudio Buttarini [2], Stefano Garetto [2], Raffaele Saladino [3], Donatella Pietrella [4], Valentina Mercati [1] and Emiliano Giovagnoni [1]

1 Aboca S.p.A., Località Aboca 20, 52037 Sansepolcro, Italy

2 Bios-Therapy, Physiological Systems for Health S.p.A., Località Aboca 20, 52037 Sansepolcro, Italy

3 Department of Chemistry and Pharmaceutical Technologies, Sapienza University of Rome, Piazzale
Aldo Moro n.5, 00185 Roma, Italy; raffaele.saladino@uniroma1.it

4 Medical Microbiology Unit, Department of Medicine and Surgery, University of Perugia, Piazzale Severi,
Building D, 4th Floor, 06129 Perugia, Italy; donatella.pietrella@unipg.it

* Correspondence: lmattoli@aboca.it

† These authors contributed equally to this work.

Summary

Figure S1. Schematic representation of the major constituent classes commonly reported for propolis. This overview is intended as a general descriptive framework; the relative proportions of these components are known to vary widely depending on botanical and geographical origin.

Figure S2. Franz diffusion cell used for assessing the resistance of the formulations' mucoadhesive properties under artificial salivary flow.

Figure S3. In vitro Transwell-based exposure model used to evaluate the barrier-forming capacity of the test samples.

Table S1. Data of samples analysed with untargeted approach

Table S2. Data of compounds analysed with targeted methods.

Table S3. Cell viability and cell viability inhibition assessed in the barrier test and internal control

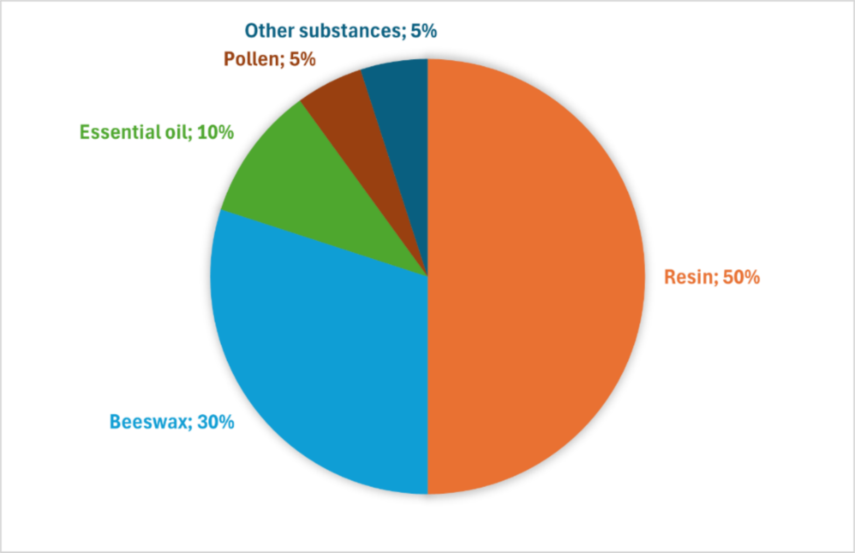

Figure S1. Schematic representation of the major constituent classes commonly reported for propolis. This overview is intended as a general descriptive framework; the relative proportions of these components are known to vary widely depending on botanical and geographical origin [1-3].

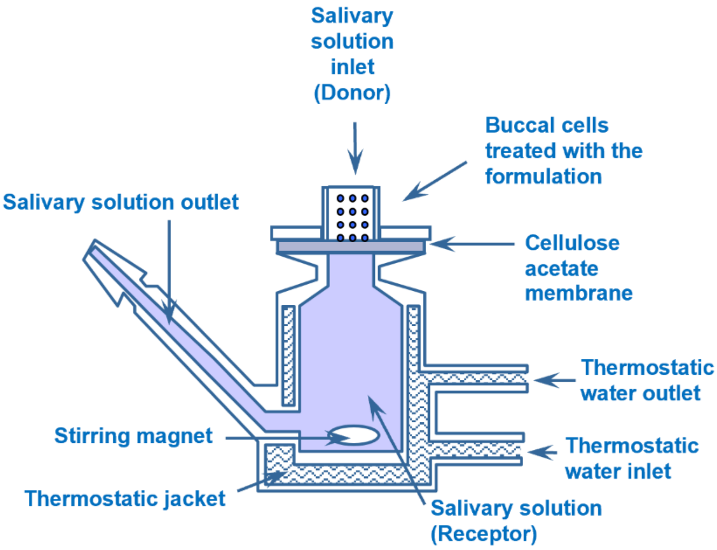

Figure S2. Franz diffusion cell used for assessing the resistance of the formulations' mucoadhesive properties under artificial salivary flow.

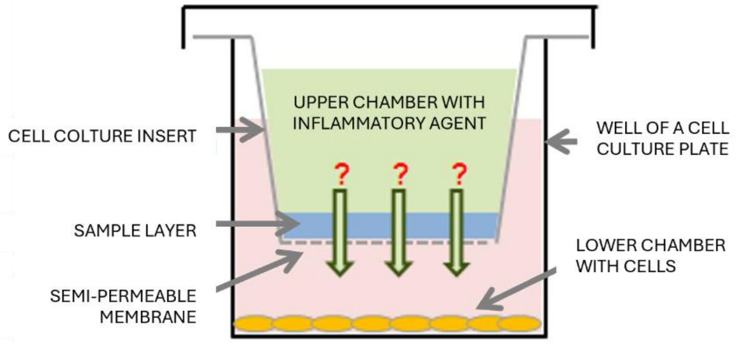

Figure S3. In vitro Transwell-based exposure model used to evaluate the barrier-forming capacity of the test samples.

Table S1. Data of samples analysed with untargeted approach

Sample cod.	Sample Description	Geographical Origin *
1-PNGE-07	*Populus nigra* dried buds	China
2-PNGE-12	*Populus nigra* dried buds	China
3-PNGE-12	*Populus nigra* dried buds	China
4-PNGE-14	*Populus nigra* dried buds	China
5-PNGE-14	*Populus nigra* dried buds	China
6-PNGE-14	*Populus nigra* female buds, freshly picked	Italy
7-PNGE-14	*Populus nigra* male buds, freshly picked	Italy
8-PNGE-14	*Populus nigra* dried buds	Italia
9-PNGE-14	*Populus nigra* dried buds	Romania
10-PNGE-14	*Populus nigra* dried buds	Romania
11-PBGE	*Populus balsamifera* dried buds	North America
12-PRO-11	Propolis	China
13-PRO-11	Propolis	Italy
14-PRO-11	Propolis	Italy
15-PRO-11	Propolis	Macedonia
16-PRO-12	Black propolis	Brazil
17-PRO-12	Green propolis	Brazil

* As declared by the supplier.

Table S2. Data of compounds analysed with targeted methods (reported in the same order of Table 1).

N°	COMPOUND	Classification	Cas N°	Molecular Formula	Molecular weight
1	4′,5-Dihydroxy-7-methoxyflavone	Flavone	437-64-9	$C_{16}H_{12}O_5$	284.26
2	Chrysin	Flavone	480-40-0	$C_{15}H_{10}O_4$	254.24
3	Naringenin	Flavone	480-41-1	$C_{15}H_{12}O_5$	272.25

4	Galangin	Flavonol	548-83-4	$C_{15}H_{10}O_5$	270.24
5	Isorhamnetin	Flavonol	480-19-3	$C_{16}H_{12}O_7$	316.26
6	Kaempferol	Flavonol	500-18-3	$C_{15}H_{10}O_6$	286.24
7	Pinobanksin	Dihydroflavonol	548-82-3	$C_{15}H_{12}O_5$	272.25
8	Pinocembrin	Flavanone	480-39-7	$C_{15}H_{12}O_4$	256.26
9	Pinostrobin	Flavanone	75291-74-6	$C_{16}H_{14}O_4$	270.28
10	3-Salicylaldehyde	Salicylate	100-83-4	$C_7H_6O_2$	122.12
11	Methyl salicylate	Salicylate	119-36-8	$C_8H_8O_3$	152.15
12	Salicin	Salicylate	138-52-3	$C_{13}H_{18}O_7$	286.28
13	Salicylic acid	Salicylate	69-72-7	$C_7H_6O_3$	138.12
14	Salicylaldehyde	Salicylate	90-02-8	$C_7H_6O_2$	122.12
15	4-Hydroxybenzoic acid	Phenolic acid	99-96-7	$C_7H_6O_3$	138.12
16	4-Coumaric acid	Phenolic acid	501-98-4	$C_9H_8O_3$	164.16
17	Gentisic acid	Phenolic acid	490-79-9	$C_7H_6O_4$	154.12
18	Protocatechuic acid	Phenolic acid	99-50-3	$C_7H_6O_4$	154.12
19	Vanillic acid	Phenolic acid	121-34-6	$C_8H_8O_4$	168.15
20	3,4-Dimethoxycinnamic acid	Hydroxycinnamic acid	14737-89-4	$C_{11}H_{12}O_4$	208.21
21	4-Methoxycinnamic acid	Hydroxycinnamic acid	830-09-1	$C_{10}H_{10}O_3$	178.18
22	4-Methoxycinnamaldehyde	Hydroxycinnamic acid	24680-50-0	$C_{10}H_{10}O_2$	162.18
23	Caffeic acid	Hydroxycinnamic acid	331-39-5	$C_9H_8O_4$	180.16
24	Caffeic acid phenethyl ester (CAPE)	Hydroxycinnamic acid	104594-70-9	$C_{17}H_{16}O_4$	284.31
25	Cinnamic acid	Hydroxycinnamic acid	140-10-3	$C_9H_8O_2$	148.16
26	Ferulic acid	Hydroxycinnamic acid	537-98-4	$C_{10}H_{10}O_4$	194.18
27	Methyl cinnamate	Hydroxycinnamic acid	103-26-4	$C_{10}H_{10}O_2$	162.18
28	α-Bisabolol	Sesquiterpene	23089-26-1	$C_{15}H_{26}O$	222.37
29	α-Curcumene	Sesquiterpene	644-30-4	$C_{15}H_{22}$	202.33
30	α-Humulene	Sesquiterpene	6753-98-6	$C_{15}H_{24}$	204.35
31	β-Eudesmol	Sesquiterpene	473-15-4	$C_{15}H_{26}O$	222.37
32	Alloaromadendrene	Sesquiterpene	25246-27-9	$C_{15}H_{24}$	204.35
33	Cedrol	Sesquiterpene	77-53-2	$C_{15}H_{26}O$	222.37
34	Guaiol	Sesquiterpene	489-86-1	$C_{15}H_{26}O$	222.37
35	Nerolidol	Sesquiterpene	7212-44-4	$C_{15}H_{26}O$	222.37

36	Valencene	Sesquiterpene	4630-07-3	$C_{15}H_{24}$	204.35
37	2-Hydroxybenzyl alcohol	Aromatic alcohol	90-01-7	$C_7H_8O_2$	124.14
38	Cinnamyl alcohol	Aromatic alcohol	4407-36-7	$C_9H_{10}O$	134.17
39	Acetophenone	Aromatic ketone	98-86-2	C_8H_8O	120.15
40	Cantharidin	Anhydride	56-25-7	$C_{10}H_{12}O_4$	196.20
41	Citric acid	Organic acid	77-92-9	$C_6H_8O_7$	192.12

Table S3. Cell viability and cell viability inhibition assessed in the barrier test and internal control

Cell group	Barrier Test – Cell viability (%)	SD	Cell viability inhibition (%)	Internal Control – Cell viability (%)	SD	Cell viability inhibition (%)
Cells tested with PBHE-SF	100.0	8.05	0	100.0	0.63	0
Positive control cells (C+)	99.36	1.10	0.64	100.0	5.07	0
Control cells	100.0	2.20	0	100.0	6.36	0

Note. PBHE concentration in PBHE-SF is 433.7 mg/mL. PBHE-SF contains 30% (v/v) ethanol, which was allowed to evaporate on the Transwell® membrane prior to cell exposure.

References

1. Burlew, R. The curious beekeeper. *Am. Bee J.* **2018**, *158*, 1155-1158. Available online: https://bluetoad.com/article/The+Curious+Beekeeper+/3195252/528323/article.html (accessed on 16 January 2026).

2. Huang, S.; Zhang, C.-P.; Wang, K.; Li, G.Q.; Hu, F.-L. Recent advances in the chemical composition of propolis. *Molecules* **2014**, *19*, 19610-19632. https://doi.org/10.3390/molecules191219610.

3. Wagh, V.D. Propolis: A wonder bees product and its pharmacological potentials. *Adv. Pharmacol. Sci.* **2013**, 308249. http://dx.doi.org/10.1155/2013/308249.
